# Pyroptosis in cancer therapy: a double-edged sword for immune activation and tumor progression

**DOI:** 10.1186/s12943-025-02506-4

**Published:** 2025-11-25

**Authors:** Ali Alishvandi, Cena Aram, Farzaneh Faraji Shahrivar, Prashant Kesharwani, Amirhossein Sahebkar

**Affiliations:** 1 Student Research Committee, Iranshahr University of Medical Sciences, Iranshahr, Iran; 2 Department of Physiology, School of Medicine, Iranshahr University of Medical Sciences, Iranshahr, Iran; 3 Department of Cell and Molecular Biology, Faculty of Biological Sciences, Kharazmi University, Tehran, Iran; 4 Department of Pharmaceutical Sciences, Dr. Harisingh Gour Vishwavidyalaya (A Central University), Sagar, Madhya Pradesh 470003 India; 5 Biotechnology Research Center, Pharmaceutical Technology Institute, Mashhad University of Medical Sciences, Mashhad, Iran; 6 Applied Biomedical Research Center, Basic Sciences Research Institute, Mashhad University of Medical Sciences, Mashhad, Iran

**Keywords:** Pyroptosis, Inflammasome, Gasdermins, Cancer immunotherapy, Tumor microenvironment, Drug resistance, Cell death signaling

## Abstract

Pyroptosis, a caspase-dependent form of inflammatory programmed cell death, is driven by inflammasome activation and gasdermin-mediated membrane pore formation. Its immunogenic nature has attracted increasing attention in oncology, particularly for overcoming therapeutic challenges such as chemoresistance, radiotherapy failure, and immune checkpoint blockade non-responsiveness. By promoting the release of pro-inflammatory cytokines and damage-associated molecular patterns (DAMPs), pyroptosis can enhance antitumor immunity and reshape the tumor microenvironment (TME). However, sustained or dysregulated pyroptosis can lead to chronic inflammation, radiotherapy-induced tissue injury, and tumorigenesis, making it a double-edged sword. Emerging evidence shows that pyroptosis exhibits cancer-type-specific roles, depending on the molecular context and the extent of activation. Therefore, understanding the molecular regulators, tumor-specific signaling, and temporal dynamics of pyroptosis is essential for its therapeutic modulation. This review comprehensively outlines the dual roles of pyroptosis in cancer progression and treatment, discusses its molecular mechanisms, and highlights recent strategies to harness or suppress pyroptosis for therapeutic gain. Targeting pyroptosis offers a promising, yet complex, avenue for immune-enhancing cancer therapies.

**Graphical abstract:**

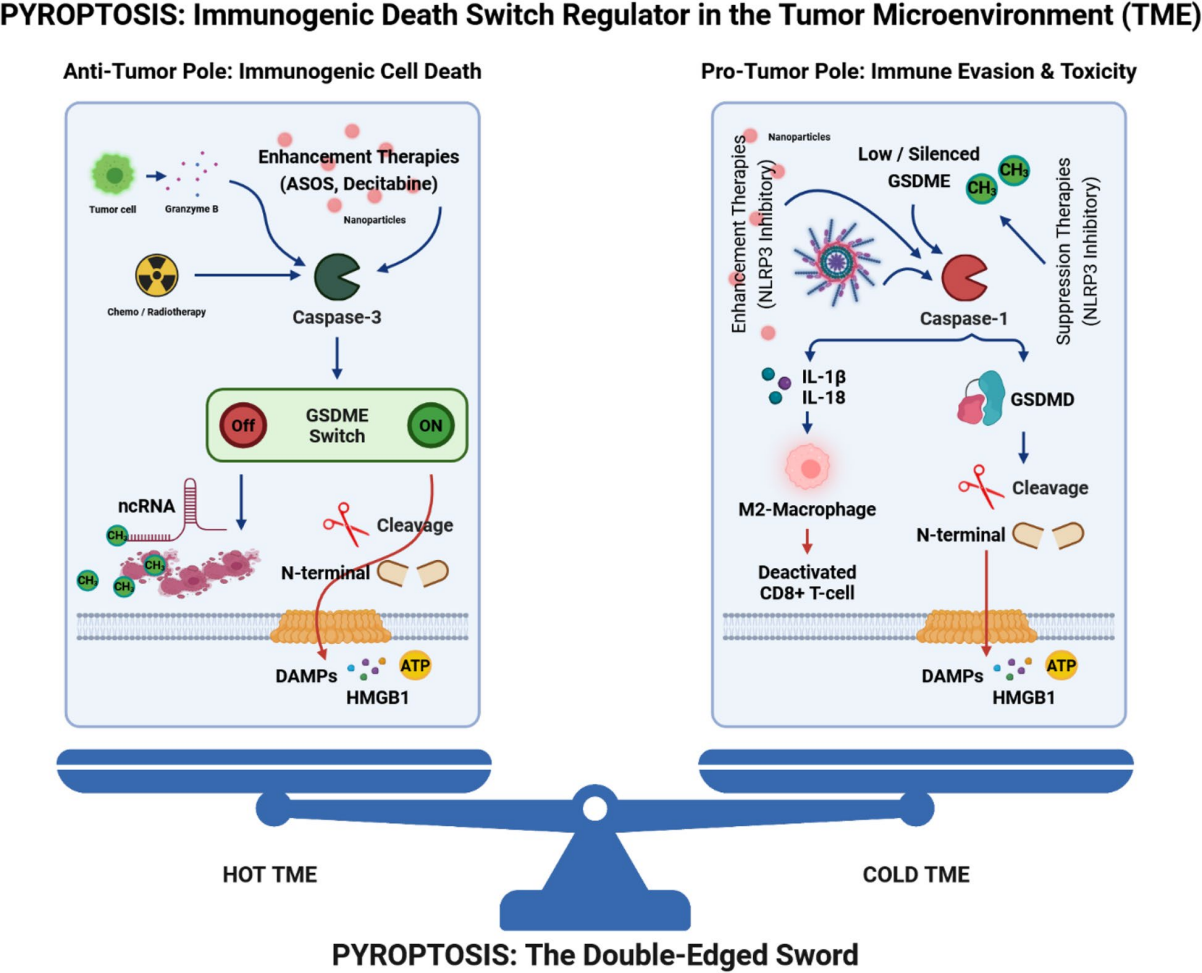

## Introduction

Despite significant advances in therapeutic approaches that target various molecular signaling pathways, cancer treatment has yet remained a complex and multifaceted dilemma [1]. Immortality, as a common feature of cancerous cells, is believed to arise from specific mutations, genetic transcriptional and post-transcriptional alterations, and protein modifications in the tumor microenvironment (TME) [2, 3]. Of note, dynamic dysregulation of immune cells in the TME, altered by genetic and epigenetic factors, serves as a key contributor to enable cells evade from programed cell death [4, 5]. Tumors employ a variety of mechanisms to avoid or restrict cell death, such as apoptosis [6], autophagy [7] and pyroptosis [8]. Among these pathways, pyroptosis, as a novel form of immunogenic and non-apoptotic programmed cell death (PCD) pathway, is mediated by a range of inflammasomes, caspases (CASPs) and the gasdermins (GSDMs) protein family [9]. The initiating role of pyroptosis in innate immunity and its connection to adoptive immunity have gained accelerating attention [10]. Moreover, as an example, recent evidence shows that the ovarian cancer A2780/CP cell line is resistant to cisplatin due to miR-221/222 overexpression and the suppression of apoptosis [11]. Therefore, the transition between pyroptosis and apoptosis or other pathways not only enhances the efficacy of treatments but also may open promising horizons for addressing chemoresistance issues to therapeutic agents [10]. It should also be noted that while pyroptosis can promote immune infiltration and improve therapeutic responses, it can also establish a chronic TME that may transform normal cells into cancerous ones, posing significant challenges [12].

In 1992, Zychlinsky and colleagues identified a form of PCD in macrophages that occurred in response to infection by the gram-negative bacterium *Shigella flexneri* [13]. Years later in 2001, D’Souza et al. appropriately named it “Pyroptosis”, emanated from the Greek words pyro (fever/fire) and ptosis (to-sis, falling), to distinguish it from the non-inflammatory PCD known as apoptosis [14]. As mentioned earlier, the role of pyroptosis in cancer is complex and depends on the type of tumor and the cellular and molecular context in which it occurs. For instance, in gastric cancer, gasdermin D (GSDMD) expression is markedly decreased, thus promoting tumor proliferation [15]. In contrast, non-small cell lung cancer (NSCLC) exhibits an upregulation of GSDMD expression, which correlates with a poor prognosis [16]. Moreover, pyroptosis can have both tumorigenic and non-tumorigenic effects by altering tumor associated macrophages (TAMs), T lymphocytes, monocytes, etc. [17]. The initiation of pyroptosis through inflammasome activation, along with the subsequent release of cytokines, can contribute to tumor progression by helping the cancer cells escape immune surveillance. However, the immune cells mobilized by pyroptosis can also activate the immune system to enhance the efficacy of cancer immunotherapy [17]. Intriguingly, recent evidence has corroborated the pivotal role of pyroptosis and its benefits in cancer immunotherapies such as chimeric antigen receptor T-cell therapy (CAR-T) and immune checkpoint inhibitors (ICIs) [10].

This review focuses on discussing the molecular mechanisms and remodeling role of pyroptosis in the TME. In addition, the alterations in pyroptosis signaling pathways across different types of cancer are described to offer new insights for identifying rational drug design targets and predicting patient clinical outcomes. Lastly, we illuminate the potential role of nanoparticles in inducing pyroptosis, as well as addressing other challenges related to drug resistance and unfavorable response rates to immunotherapy approaches.

## Molecular mechanisms of pyroptosis

**Fig. 1:**
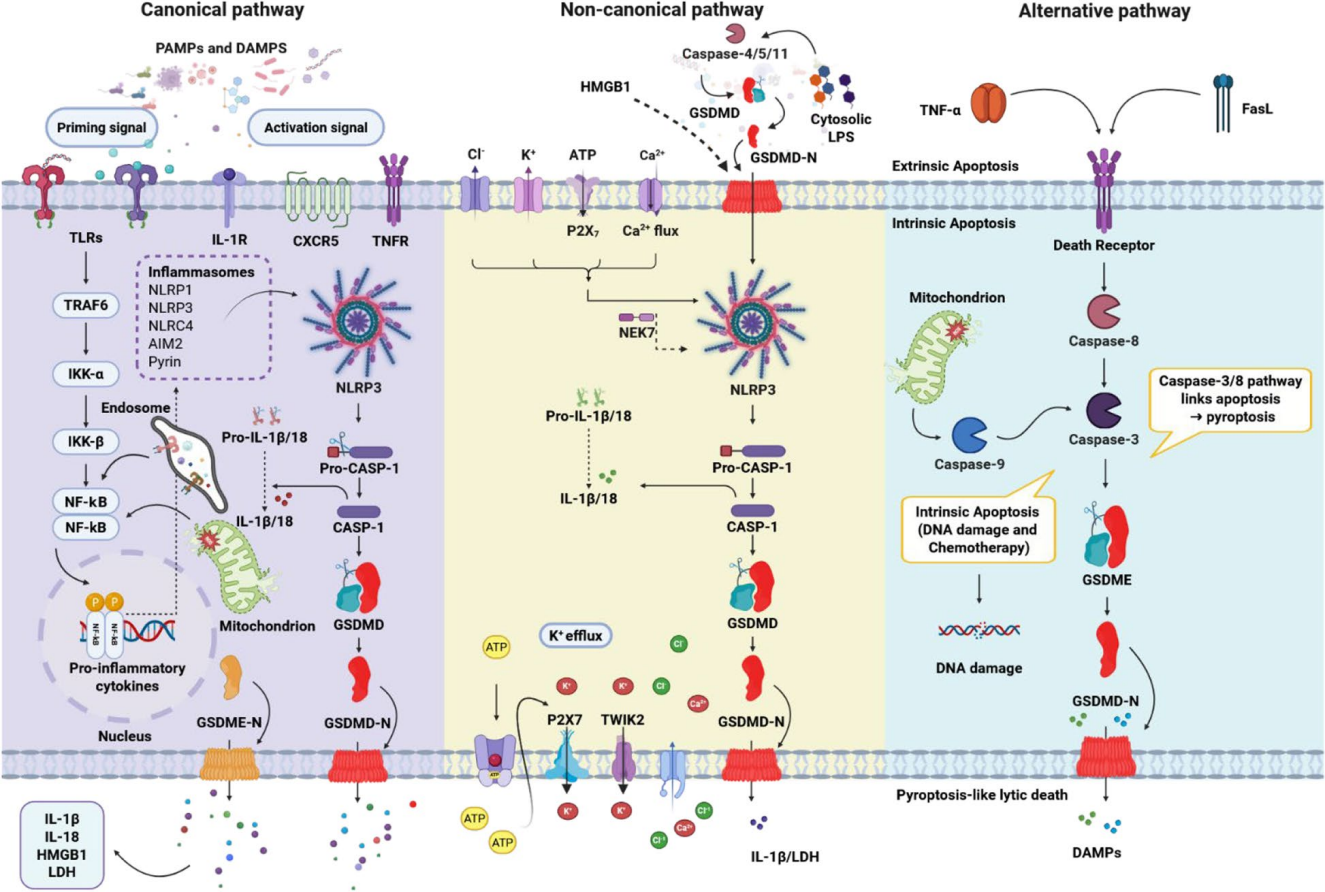
The currently identified molecular signaling pathways that lead to pyroptosis

Pyroptosis can proceed through multiple signaling routes, of which the canonical and non-canonical inflammasome pathways remain the most extensively characterized (Table 1) [9, 23]. In the canonical pathway, pathogen-associated molecular patterns (PAMPs) or damage-associated molecular patterns (DAMPs) are recognized by specific cytosolic inflammasome sensors, leading to caspase-1 activation, gasdermin-mediated membrane pore formation, and the release of pro-inflammatory cytokines such as IL-1β and IL-18. In contrast, the non-canonical pathway is initiated by direct binding of intracellular lipopolysaccharide (LPS) to caspase-4/5 (humans) or caspase-11 (mice), triggering gasdermin cleavage without the requirement for priming. Although these pathways differ in their initiating signals and upstream adaptors, both culminate in loss of membrane integrity and inflammatory mediator release. Understanding these mechanisms in cancer is essential, as they influence tumorigenesis, immune evasion, and the re-shaping TME, offering potential therapeutic leverage points. Figure 1 provides an integrated overview of the major pyroptotic pathways, including canonical, non-canonical, and apoptosis-linked “alternative” routes, highlighting key triggers, molecular components, and execution phases.

**Table 1:** Integrated summary of canonical and noncanonical inflammasome pathways and their downstream pyroptosis executors in cancer

Pathway/Inflammasome	Key triggers	Core components	Key outputs	Implications in cancer	Reference
NLRP3	K⁺ efflux (P2X7/ATP), ROS, cardiolipin, lysosomal rupture, crystals, metabolic stress	NLRP3/ASC/pro-caspase-1	IL-1β, IL-18, GSDMD cleavage → pyroptosis	Most extensively studied; context-dependent; in many tumors NLRP3 → chronic IL-1β-driven inflammation, angiogenesis and metastasis; in selected settings NLRP3 activation can enhance anti-tumor immunity (depends on cell type, stage, TME)	[18–20]
AIM2	Cytosolic dsDNA (pathogen- or host-derived; DNA damage)	AIM2 (HIN-200 domain protein)/ASC/pro-caspase-1	IL-1β, IL-18, pyroptosis; DNA-sensing effects	Often tumor-suppressive in epithelial cancers (e.g., CRC, some breast cancer studies) via limiting proliferation and promoting immune surveillance; but can be pro-tumor in particular contexts (e.g., some glioblastoma reports)	[18, 20]
NLRC4	Bacterial ligands via NAIPs (flagellin, T3SS proteins)	NAIP(s) + NLRC4 (CARD)/caspase-1	IL-1β/IL-18, pyroptosis	Less data than NLRP3; evidence for immune-surveillance and anti-tumor roles in GI cancers (CRC); inflammasome-independent immune reprogramming also reported; promising but still preclinical-heavy	[18]
NLRP1	Proteotoxic/ribotoxic stress, bacterial proteases, DPP8/9 inhibitors	NLRP1 (FIIND, CARD)/ASC/pro-caspase-1	IL-1β, IL-18, pyroptosis	Prominent in barrier tissues; evidence for protective roles in some epithelial contexts and genetic links to skin cancer syndromes; clinical data limited; activation mechanisms differ in human vs. mouse (important translational caveat)	[18, 21, 22]
GSDME	Cleaved by caspase-3, granzyme B	GSDME (pore-forming N-term fragments)	Plasma membrane pores, lytic death, DAMP release, immune activation	Mostly pro-immunogenic; enhances therapy response but may induce CRS in CAR-T context	

*Abbreviations: NLRP3* NOD-, LRR- and pyrin domain-containing protein 3, *AIM2* Absent in melanoma 2, *NLRC4* NLR family CARD domain-containing protein 4, *NLRP1* NLR family pyrin domain-containing 1, *NAIP* NLR family apoptosis inhibitory protein, *CARD* Caspase recruitment domain, *FIIND* Function-to-find domain, *ASC* Apoptosis-associated speck-like protein containing a CARD, *GSDME* Gasdermin E, *IL-1β* Interleukin-1 beta, *IL-18* Interleukin-18, *DAMP* Damage-associated molecular patterns, *TME* Tumor microenvironment, *CRC* Colorectal cancer, *T3SS* Type III secretion system, *ROS* Reactive oxygen species, *ATP* Adenosine triphosphate, *CRS* Cytokine release syndrome

### Canonical pathway

**Fig. 2:**
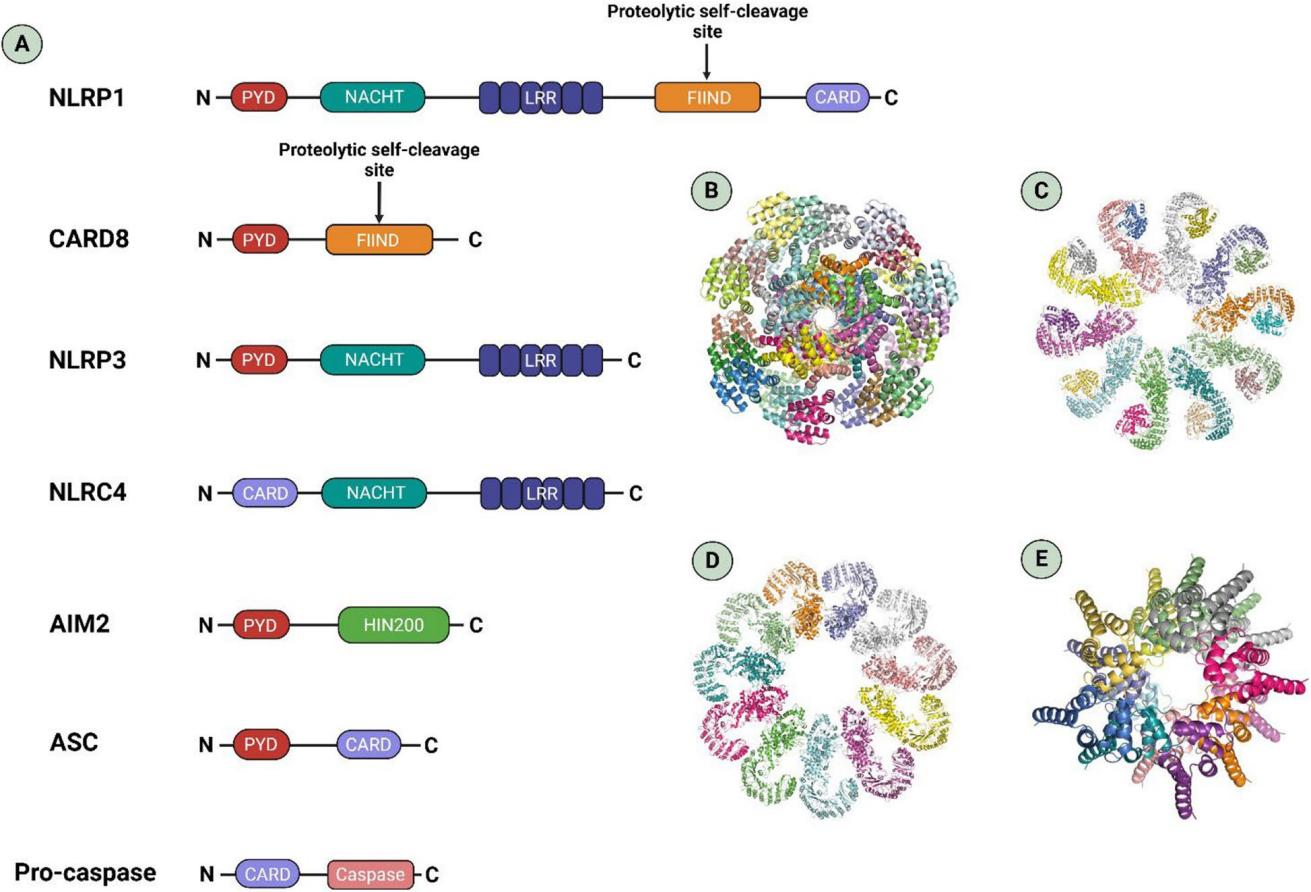
Domain architecture and interaction of inflammasome proteins involved in pyroptosis. **A** Depicts the structural subunits of inflammasomes and their respective components coming together to form complexes. **B**, **C**, **D**, **E** Derived from Protein Data Bank (PDB) structures, illustrating how these proteins assemble to form NLRP1, NLRP3, NLRC4, and AIM2, respectively

To date, five major inflammasome-forming sensors including NLRP1, NLRP3, NLRC4, pyrin, and AIM2 are recognized as central initiators of this process [24]. These receptors assemble into multiprotein complexes, with the individual components depicted in Fig. 2. Upon activation, the assembled inflammasome converts pro-caspase-1 into its active form, leading to GSDMD cleavage and pore formation, and the maturation of IL-1β and IL-18, which collectively drive lytic cell death and inflammatory signaling [24] In terms of the domain architecture of inflammasomes, including their PYD- and CARD-mediated interactions with ASC and pro-caspases, please see Fig. 2. These domain differences influence adaptor dependency and modulate the amplitude of inflammasome signaling in different cellular contexts.

#### NLRP1–ASC–caspase-1 complex pathway

Among canonical inflammasome sensors, NLRP1 is distinguished by the presence of a unique function-to-find (FIIND) domain that undergoes autoproteolytic cleavage, generating two non-covalently associated fragments [25] (Fig. 2A). Ligand recognition by NLRP1 triggers conformational changes and leads to oligomerization of NACHT domain. Downstream, active caspase-1 cleaves GSDMD, forming membrane pores that facilitate IL-1β and IL-18 maturation and release [26]. Besides GSDMD, which initiates pore formation following inflammasome activation, Zhou and Abbott [26] showed that GSDME can be activated later to mediate cytokine release, particularly IL-1β, during a “sublytic phase”. In this phase, pores are formed without causing full cell lysis. This process requires higher presence of GSDME and significant pore assembly (Fig. 1).

#### NLRP3–ASC–caspase-1 complex pathway

The NLRP3 inflammasome follows an activation scheme broadly similar to NLRP1 but is distinguished by a requisite two-phase process comprising priming and activation. In the priming phase, danger or pathogen signals engage cell-surface or endosomal receptors, activating the NF-κB transcriptional program via adaptor proteins, resulting in upregulation of NLRP3 and pro-IL-1β/IL-18 expression [27–31]. The activation phase is subsequently triggered by diverse intracellular stress cues, including mitochondrial reactive oxygen species (ROS), leaked mtDNA, and lipids like cardiolipin (Table 1) [32]. Mitofusin 2 and mitochondrial antiviral signaling protein (MAVS) cooperate in positioning NLRP3 at mitochondria–endoplasmic reticulum contact sites, facilitating efficient inflammasome assembly. Once activated, caspase-1 mediates GSDMD cleavage, pore formation, and cytokine maturation, initiating potent inflammatory signaling [31, 32] (Fig. 1). Beyond its well-established role in infection-driven inflammation, NLRP3 has profound cancer relevance: recent studies have shown that NLRP3 activation in tumor-associated macrophages can amplify immunosuppressive myeloid cell infiltration in pancreatic cancer [33], while in other contexts, such as melanoma, NLRP3-dependent IL-1β release enhances CD8⁺ T-cell recruitment to the tumor bed [34]. Related priming/activation phases are shown in Fig. 1. A recent study has expanded the conceptual framework of NLRP3 function in cancer beyond its canonical inflammasome activity. Accogli et al. revealed that intrinsic expression of NLRP3 in Th17 cells sustains their protumorigenic and immunosuppressive phenotype through an inflammasome-independent pathway. Mechanistically, NLRP3 was shown to interact directly with the TGF-β receptor, promoting mothers against decapentaplegic homolog 3 (SMAD3) phosphorylation and facilitating Th17-to-Treg trans-differentiation within the tumor microenvironment. Loss of NLRP3 disrupted this process, leading to heightened Th17 inflammatory output (IFN-γ, Granzyme B, TNF-α) and restored CD8⁺ T-cell cytotoxicity, ultimately constraining tumor growth [35]. This study underscores that NLRP3 not only orchestrates innate immune sensing but also acts as a molecular switch controlling adaptive immune plasticity, redefining its relevance in the evolving crosstalk between inflammation and cancer immunity.

#### AIM2–ASC–caspase-1 complex pathway

The AIM2 inflammasome is unique among canonical sensors for its direct recognition of cytosolic double-stranded DNA (dsDNA) via a positively charged HIN-200 domain, paired with an N-terminal pyrin domain (PYD) [36]. Upon dsDNA recognition via HIN domain, AIM2 recruits ASC to activate caspase-1 and subsequent gasdermin cleavage (Fig. 2A). AIM2 activity is modulated by immune regulators: type I interferon (IFN-1) enhances its expression, thereby amplifying caspase-1-mediated responses, while p202, another PYHIN family member, can competitively inhibit AIM2–DNA binding and suppress downstream activation [37]. Dysregulated AIM2 function has implications beyond infection control; recent oncological studies reveal that decreased AIM2 expression in colorectal and breast tumors correlates with increased inflammatory cytokine milieu and poor prognosis [38], whereas in glioblastoma, AIM2 expression is markedly elevated and plays a role in impairing mitochondrial function [39]. This duality reinforces AIM2’s status as both a driver of chronic inflammation-related tumorigenesis and a facilitator of beneficial immune activation, depending on tumor context and therapeutic strategy. For an integrated overview of pyroptosis components and their context-dependent pro- or antitumor roles, see Table 2. Recent evidence highlights a critical role of AIM2-like receptors (ALRs) beyond innate immunity, revealing their involvement in genotoxic tissue injury. Jiang et al. demonstrated that mice deficient in ALRs exhibit resistance to irradiation-induced bone marrow damage, indicating a direct contribution of these receptors to radiation sensitivity. Mechanistically, nuclear ALRs inhibit DNA repair by binding to chromatin and preventing its decompaction, thereby promoting genome instability, micronuclei formation, and cell death [108]. Importantly, this function is independent of their canonical immune role, uncovering ALRs as potential therapeutic targets to mitigate radiation-induced tissue injury and as biomarkers for predicting outcomes of radio- or chemotherapy (readers are referred to "Radiation therapy" section).

**Table 2:**
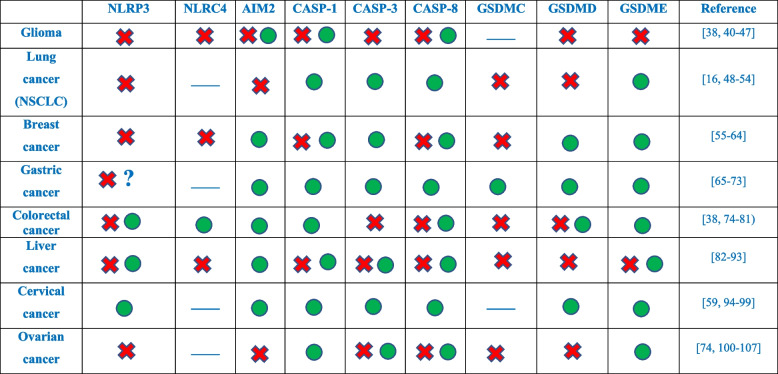
Pro- and anti-tumor roles of key pyroptosis and apoptosis regulators across different cancers (pro-tumor:; anti-tumor:) [16, 38, 40–107]

#### NLRC4/NLRP1b (± ASC)–caspase-1 complex pathway

The inflammasome sensors NLRC4 and also murine NLRP1b display notable structural and functional differences compared to NLRP3 or AIM2. Given that their structures do not contain a PYD domain in their structures, which is required for binding with the PYD domain of ASC, they are triggered directly without recruiting ASC. It should be noted that in the presence of ASC, the specks formed by ASC still play a promoting role in the activation of NLRC4 and NLRP1b (Table 1) [109]. NLRC4 is triggered in response to the bacterial flagellin or type III secretion system (T3SS). Recently, Churchill et al. revealed that the NLRC4 activation in intestinal epithelial cells (IECs) leads to the secretion of prostaglandin E2 and IL-18 [110]. The NLRC4 inflammasome assembles upon recognition of cytosolic bacterial ligands by NLR family Apoptosis Inhibitory Proteins (NAIPs), leading to conformational changes in NLRC4 (Table 1). This activation results in the recruitment and cleavage of pro-caspase-1, which in turn mediates GSDMD-dependent pyroptosis and the maturation of IL-1β and IL-18. In addition, NLRC4 can engage caspase-8, subsequently activating caspase-3 and −7 to trigger apoptosis [111]. It appears that these complexes may have more NLRC4 in comparison to each NAIP, and these NLRC4 inflammasomes are able to activate pro-caspase-1 either directly without recruiting ASC or via an ASC adaptor [112, 113].

### Non-canonical pathway

Intracellular LPS signals initiate the non-canonical pathway through direct binding to pro-caspase-4/5 in humans (or its murine homolog, pro-caspase-11) [114]. Of note, caspase-4/5/11, unlike caspase-1, are not directly capable to cleave pro-IL-1β/18 into the mature state. Instead, they are essential for the maturation and secretion process of proinflammatory components by promoting NLRP3/caspase-1 pathway [115, 116]. Ion fluxes (K⁺, Ca^2^⁺, Na⁺, and Cl⁻) are now widely recognized as key drivers of NLRP3 inflammasome activation (Table 1) [117]. Upon caspase-11 auto-cleavage, pannexin-1 channels open, releasing extracellular ATP that serves as a DAMP to activate P2X7 receptors [118]. Di et al. [119] demonstrated that P2X7 and the Tandem of pore-domains in a Weakly Inward rectifying K^+^ channel 2 (TWIK2) in macrophages act in concert: P2X7 stimulation increases Ca^2^⁺ and Na⁺ influx, depolarizing the membrane and thereby promoting rapid K⁺ efflux through TWIK2, which is a critical event for NLRP3/caspase-1 activation. NIMA-related kinase 7 (NEK7) further facilitates this process by binding to the LRR and NACHT domains of NLRP3 to promote oligomerization, though it is dispensable for NLRC4- and AIM2-driven inflammasome assembly [120]. In parallel, Cl⁻ efflux via volume-regulated anion channels (VRACs) also contributes to NLRP3 activation, and interestingly, non-steroidal anti-inflammatory drugs (NSAIDs) have been shown to block VRACs and attenuate this pathway [121]. Together, these findings highlight a coordinated ionic network that primes and activates the NLRP3 inflammasome, although the precise molecular coupling among these ion fluxes remains to be clarified. The high-mobility group box 1 (HMGB1), is also considered another pyroptotic component, although the literature conflicts on how it contributes to the activation process. HMGB1 is thought to bind to LPS and transport it into macrophage cytosol, leading to the activation of caspase-11 and caspase-1 [122] (Fig. 1). Generally, caspase-4 shares similarities with caspase-1, as cleaving both GSDMD and pro-IL18. Nevertheless, caspase-4 lacks the ability to split and activate pro-IL-1β. On the contrary, caspase-5 is able to cleave GSDMD but limited efficacy in processing immature form of cytokines [123].

Of note, a study has suggested potential links between non-canonical pyroptotic pathways and cancer-relevant caspases. Yang et al. demonstrated that in neuroblastoma cells, NF-κB signaling upregulates caspase-4, which is required for Fas-induced apoptosis. Caspase-4 acted downstream of caspase-8 and upstream of PARP and caspase-3, highlighting its role as a key mediator of pro-apoptotic signaling [124]. Although traditionally associated with inflammatory responses and bacterial infection, caspase-4’s regulation by NF-κB suggests it could intersect with gasdermin-mediated pyroptosis, providing a mechanistic rationale for exploring non-canonical pathways in cancer contexts, particularly through GSDMD activation.

### Caspase-3/8–mediated pyroptosis

Pyroptotic signaling can intersect with apoptosis through the actions of caspase-3 and caspase-8, which cleave specific gasdermin isoforms to switch cell fate from non-lytic apoptosis to inflammatory cell death [125, 126]. This crossover is exemplified by the pathogen *Yersinia* via its effector YopJ, which inhibits TGFβ-activated kinase 1 (TAK1) and Iκβ kinase β (IKKβ), thereby promoting receptor-interacting protein kinase 1 (RIPK1)- caspase-8 activation. Active caspase-8 cleaves GSDMD and GSDME, forming membrane pores and initiating pyroptosis [127, 128]. Chemotherapeutic drugs can either induce pyroptosis or shift apoptosis towards caspase-3/GADME-mediated pyroptosis [129–131]. Of note, low levels of GSDME make cells more susceptible to apoptosis, while high levels promote pyroptosis [126]. Additionally, in esophageal cancer treated by cisplatin, an identified mechanism of action involves the activation of calpain-1/2 (CAPN1/2)/Bcl-2 homologous antagonist/killer (BAK)/Bcl-2-associated X protein (BAX)/caspase-9/caspase-3/GSDME signaling axis [132]. Furthermore, activation of caspase-9 in cancer cells treated with MEK, EGFR, or ALK inhibitors (e.g., trametinib, erlotinib, ceritinib) can lead to caspase3-mediated pyroptosis [133]. Recently, Hou et al. elucidated a non-immune checkpoint function for nuclear programmed death-ligand 1 (PD-L1), which can switch the conversion of apoptosis caused by TNF-α to pyroptosis in cancerous cells. They discovered that in low-oxygen environments, p-Stat3 directly interacts with PD-L1 and promotes its translocation to the nucleus. This process leads to increased transcription of GSDMC, which is subsequently cleaved by caspase-8 [55]. Altogether, caspase-3/8-mediated pyroptosis acts as a leverage to regulate the transition between apoptosis and pyroptosis, allowing for adjustment according to therapeutic goals. For a comparative summary of key pyroptosis regulators and their dual oncological roles, refer to Table 2.

### Granzyme-mediated pyroptosis

Granzymes are a type of serine proteases mainly secreted by natural killer cells (NK) and cytotoxic T lymphocytes (CTLs), where they trigger cell death in virus-infected cells or tumor cells. These granzymes enter the target cells through pores created by perforin proteins. Granzyme A and B have different mechanisms of action: both may induce pyroptosis independently of caspases, but granzyme B can also recruit caspases to trigger pyroptosis. The caspase-independent pathway is discussed in the next subsection.

Research indicates that GSDME plays a crucial part in sensitizing tumor cells to poly (ADP-ribose) polymerase inhibitors (PARPi), suggesting a potential avenue to enhance therapeutic efficacy in resistant cancers. On the one hand, PARPi treatment triggers GSDMC/caspase-8-mediated pyroptosis. On the other hand, T cell-derived granzyme B activates caspase-6 to cleave GSDMC, implying that combining PARPi with immune checkpoint inhibitors could synergistically amplify tumor clearance. Moreover, IFN-γ upregulates GSDMC expression, further strengthening cytotoxic T cell–driven pyroptosis and underscoring its potential as a predictive biomarker of immunotherapy responsiveness [134]. Liu et al. demonstrated that CAR T cells induce caspase-3/GSDME-mediated pyroptosis through the release of granzyme B in B cell acute lymphoblastic leukemia [135]. In HBV-related acute-on-chronic liver failure, the caspase-8/GSDMD pathway activated by granzyme B is responsible for inducing neutrophil extracellular traps and disease progression [136], illustrating that the same pyroptotic axes may yield beneficial or detrimental outcomes depending on the immune context.

### Caspase-independent pathway

Over the last few years, several elements have come to light that induce pyroptosis independently of caspase recruitment. Granzyme A cleaves GSDMB predominantly at Lys^244^ and has the ability to convert apoptosis into pyroptosis by additional expression of granzyme A-cleavable GSDMB [137]. Moreover, it has been shown that granzyme B can directly activate GSDME at the same location that is affected by caspase-3 [138].

Kambara et al. revealed that GSDMD cleavage and its activation in response to *Escherichia coli* is independent of caspases and mediated by the neutrophile serine protease, ELAstase-Neutrophil Expressed (ELANE). Intriguingly, GSDMD deficiency gives rise to persistence and accumulation of neutrophiles, thereby establishing an augmented host response to *Escherichia coli*. Additionally, depending on the context, GSDMD can act as either an anti-inflammatory or proinflammatory factor. In *Escherichia coli*-induced peritonitis, GSDMD deficiency expedites neutrophile death, leading to their engulfment and clearance by macrophages, thereby exerting an anti-inflammatory effect [139]. Furthermore, the Zika virus (ZIKV) protease can directly process GSDMD in a way that does not rely on caspases [140]. The divinyl sulfone compound demonstrates a similar GSDMD cleavage pattern independent of caspases in hepatocyte cells, with a large accumulation of ROS, hypothesized to be a key contributor to this process [141]. Streptococcal pyrogenic exotoxin B (SpeB), a virulence factor of *streptococcus pyrogens*, stimulates GSDMA-mediated pyroptosis in keratinocytes. Research indicates that GSDMA not only acts as a substrate but also functions as a simple one-molecule sensor of SpeB, facilitating caspase-independent pyroptosis. Notably, SpeB-triggered pyroptosis is completely blocked by cysteine protease inhibitors, but not by caspase inhibitors [142, 143] (Fig. 1). Another recent study found that a sudden drop in ATP levels during irreversible electroporation causes a type of cell death that doesn’t rely on caspases. However, it remains unclear whether this can be attributed to pyroptosis pathway [144]. Overall, our knowledge of caspase-independent pathways is limited and requires further research and clarification.

## Interplay between pyroptosis and other cell death mechanisms

Pyroptosis, apoptosis, and necroptosis are co‐present in many settings, and the boundaries between them are more porous than often assumed. At their cores, apoptosis, necroptosis, and pyroptosis differ in triggers, effectors, and inflammatory consequences. Apoptosis is non-lytic and generally immunologically silent, executed by caspase-3/7 downstream of intrinsic (mitochondrial) or extrinsic (death receptor) pathways. Necroptosis and pyroptosis are lytic. In terms of inflammatory processes, in general, necroptosis depends on RIPK1/RIPK3 activation of mixed lineage kinase domain-like protein (MLKL), and pyroptosis is driven by inflammatory caspases (e.g., caspase-1, −4/5/11), cleaving GSDMs to form membrane pores [145].

Yet, multiple “cross‐wires” exist that allow one death modality to influence or transform into another. For example, caspase-3, conventionally an apoptotic executioner, can cleave GSDME, converting an apoptotic stimulus into GSDME-mediated secondary pyroptosis [146]. Caspase-8, long known for initiating the extrinsic apoptotic cascade, also functions as a molecular switch: when inhibited or under certain contexts, its suppression can permit RIPK1/RIPK3/MLKL–driven necroptosis; conversely, caspase-8 has been implicated in direct cleavage of GSDMD, thus bridging apoptotic and pyroptotic pathways [145]. Moreover, Wu et al. revealed that caspase-8 inactivation in myeloid cells induces autophagy-dependent inflammasome activation even when classical apoptosis and necroptosis pathways are blocked. Their study demonstrated that caspase-8, caspase-1/11, and GSDMD are essential for this atypical inflammasome-mediated cell death, whereas NLRP3 and RIPK1 are dispensable, and highlighted the critical role of the autophagy–cathepsin B axis in mediating this response [147]. The recently described concept of PANoptosis, a unified cell death modality combining features of pyroptosis, apoptosis, and necroptosis via a multiprotein PANoptosome complex offers a conceptual scaffold for how cells may flexibly deploy multiple death modules in concert [148].

Of note, these pathways also show mechanistic convergences. Both GSDMD pores and MLKL channels produce ionic fluxes (notably K⁺ efflux), which can secondarily activate the NLRP3 inflammasome and bolster further inflammatory signaling, thus creating positive feedback loops between necroptosis and pyroptosis [149]. Indeed, in some contexts where pyroptotic machinery is disabled, cells may default or be coerced into necroptosis, and vice versa [149]. Temporal ordering also diverges: membrane permeabilization is an earl**y** hallmark in pyroptosis, whereas in necroptosis the sub-lytic phase may persist until MLKL oligomerization eventually breaches membrane integrity [150]. Yet these overlaps generate interpretative risks. Many experimental systems rely on genetic knockouts or pharmacologic inhibitors, which may force cells into alternate death fates that would not be physiologically relevant in intact organisms. Likewise, morphological criteria (blebbing, swelling, membrane rupture) are often insufficient to distinguish among death types in situ [151].

From a translational vantage, the crosstalk is double-edged. In cancer therapy, where apoptosis resistance is pervasive, redirecting cell death toward pyroptosis or necroptosis is attractive: it can enhance immunogenic cell death and promote anti‐tumor immunity [152]. However, indiscriminate activation of lytic death pathways in nonmalignant tissue risks exacerbating inflammation, tissue injury, or cytokine storm. Second, the redundancy implies that inhibition of one pathway (e.g., blocking caspases) might backfire by aggravating necroptosis or triggering PANoptosis. Rigorous biomarker strategies are lacking to discriminate death modes in patient tissues, complicating clinical translation. While the canonical versus noncanonical distinction in pyroptosis remains a useful organizing principle, real cells often operate at the interface of multiple death modalities. Therefore, cautiously interpreting various crosstalk is essential both for mechanistic rigor and for translational ambition.

## The role of pyroptosis in cancers

In the human genome, an estimated 75% of genes undergo transcription into RNA, while a mere 3% is transcribed into messenger RNAs (mRNAs) encoding proteins. Predominantly, genetic, transcriptional and post-transcriptional alterations can give rise to aberrant expression of proteins and non-coding RNAs (ncRNAs) in the context of cancers. However, ncRNAs such as microRNAs (miRNAs) and long non-coding RNAs (lncRNAs) are unable to produce protein; they have an important regulatory function in modulating the activation or silence cell signaling pathways (Table 3) [201–204]. The involvement of ncRNAs in cancer as well as their diagnostic, prognostic and therapeutic applications have been extensively explored and reviewed [205–211]. In this section, we review the footprints of pyroptosis, including the upregulation and downregulation of its key signaling components, with the aim of gaining valuable insights for applying them to the field of drug design and targeted therapy as well. This section focuses on highlighting the expression behavior of pyroptosis signaling pathways. Further discussion on the role of drugs in modulating these pathways will follow in the next section. As summarized in Table 2, pyroptosis exerts dual and context-dependent roles across major cancer types. In glioma, lung, breast, gastric, colorectal, and other malignancies, distinct gasdermins, inflammasomes, and upstream regulators can either promote tumor progression through chronic inflammation, immune escape, and enhanced invasion or suppress growth by triggering immunogenic cell death, restoring chemosensitivity, and stimulating anti-tumor immunity. For instance, upregulation of GSDMD in lung cancer or GSDMC in colorectal cancer correlates with poor prognosis and therapeutic resistance, whereas reactivation of GSDME in breast cancer or AIM2 in gastric cancer has been shown to re-sensitize tumors to chemotherapy and inhibit proliferation. The evidence compiled in this table highlights that pyroptosis is not universally beneficial or detrimental but rather highly context-specific, reinforcing the need for precision strategies that enhance its anti-tumor effects while mitigating its tumor-promoting potential.

**Table 3:** Identified lncRNAs in pyroptosis

Long non-coding RNAs	Expression	Target/regulatory axis	Function	Type of cancer	References
XIST	Upregulated	miR-335/SOD2/ROS/NLRP3	Inhibits pyroptosis, promotes cell proliferation	Non-small cell lung cancer	[153]
ADAMTS9-AS2	Downregulated	miR-223-3p/NLRP3	Inhibits tumor progression, sensitizes cells to cisplatin	Gastric cancer	[154]
RP1-85F18.6	Upregulated	ΔNp63/GSDMD	Promotes the growth, invasion, metastasis, and interferes with normal cell cycle; suppresses apoptosis and pyroptosis	Colorectal cancer	[155]
SNHG7	Upregulated	miR-34a/SIRT1	Inhibits NLRP3-dependent pyroptosis	Hepatocellular carcinoma	[156]
MALAT1	Upregulated	miR-124/SIRT1	Cancer cell progression	Cervical cancer	[157]
LRRC75A-AS1	Upregulated	IGF2BP1/SYVN1/NLRP3/Smad2/3	Drives epithelial-mesenchymal transition and promotes pyroptosis	Cervical cancer	[158]
HOTTIP	Upregulated	miR-148a-3p/AKT2	Inhibits pyroptosis, increases cell proliferation	Ovarian cancer	[159]
MEG3	Downregulated	NLRP3/caspase-1/GSDMD	Enhances cisplatin efficacy by promoting pyroptosis	Triple-negative breast cancer	[160]
TCONS-14036	Downregulated	miR-1228-5p/PRKCDBP	Its downregulation reduces pyroptosis	Non-small cell lung cancer	[161]
LINC00969	Upregulated	NLRP3/caspase-1/GSDMD	Promotes acquired gefitinib resistance	Lung cancer	[162]
LINC00511	Upregulated	hsa-miR-573	Poor prognosis; influences tumor immune infiltration	Breast cancer	[163]
MIR193BHG	Upregulated	Not specified	Promotes cell growth, invasion, and migration	Lung squamous cell carcinoma	[164]
NEAT1	Upregulated	miR-448/GSDME	Regulates radioresistance; enhances pyroptosis	Colorectal cancer	[165]
GAS5	Downregulated	ASC/caspase-1	Suppresses cancer progression by promoting inflammasome formation	Ovarian cancer	[166]
HOXC-AS2	Upregulated	miR-876-5p/HKDC1	Controls the development of cancer and pyroptosis in a microenvironment with high glucose levels surrounding tumors	Endometrial cancer	[167]
ZNF674-AS1	Upregulated	IGF2BP3/CA9	Drives cell growth, inhibits cisplatin-induced pyroptosis	Neuroblastoma	[168]
LINC01133	Upregulated	miR-30b-5p/SIRT1	Promotes tumor growth, inhibits pyroptosis	Pancreatic adenocarcinoma	[169]
DANCR	Upregulated	miR-135a/NLRP3	Promotes cell proliferation and invasion	Pancreatic cancer	[170]
RGMB-AS1	Upregulated	miR-22/NLRP3	Promotes cell proliferation and invasion	Laryngeal squamous cell carcinoma	[171]
miR-214	Downregulated	Caspase-1 NLRP3	Inhibits cellular proliferation and migration Cellular proliferation	Glioma cancer Cervical cancer	[172] [94]
miR-335	Downregulated	ZNF-148/miR-335/SOD2/ROS	Mediates oxidative stress and pyroptosis	Breast cancer	[173]
miR-182	Upregulated	STAT5/miR-182/NLRP3	Mediates pyroptosis, Reduces proliferation and colony formation	Breast cancer	[174]
miR-200b	Downregulated	miR-200b/JAZF1/NF-κB	Mediates pyroptosis, promotes cell growth	Breast cancer	[175]
miR-1290	Upregulated	NLRP3	Increases radioresistance by inhibiting pyroptosis	Triple-negative breast cancer	[176]
miR-155	Upregulated	NLRP3	Promotes cell proliferation, migration, inflammation; inhibits apoptosis	Triple-negative breast cancer	[177]
miR-155-5p	Upregulated	GSDME	Promotes cetuximab resistance; inhibits apoptosis and pyroptosis	Triple-negative breast cancer	[178]
miR-21-5p	Upregulated	TGFBI	Induces pyroptosis	Colorectal cancer	[179]
miR-1246	Upregulated	NLRP3	Promotes tumor progression by disrupting CD8 + T cell infiltration and function	Colorectal cancer	[180]
miR-223	Downregulated	NLRP3	Post-translational suppression of NLRP3; induced autophagy; enhanced cytokine release	Colorectal cancer	[181]
miR-497	Downregulated	PELP1	Regulates pyroptosis towards cancer elimination	Esophageal squamous cell carcinoma	[182]
miR-93-5p	Upregulated	GSDME	Promotes proliferation and migration; enhances ionizing radiation resistance	Non-small cell lung cancer	[183]
miR-196b-5p	Upregulated	ING5	Enhances T cell pyroptosis, promotes tumor progression	Non-small cell lung cancer	[184]
miR-556-5p	Upregulated	NLRP3	Inhibits cisplatin sensitivity and promotes chemoresistance	Non-small cell lung cancer	[185]
miR-223-3p	Downregulated	NLRP3	Reduces cell growth and migration	Glioblastoma multiform	[186]
miR-145	Downregulated	GSDMD	Inhibits cell proliferation and induces pyroptosis	Cervical cancer	[97]
MiR-624-5p	Upregulated	NLRP3/EMT/IL-1β/Wnt/β-catenin	Enhances gemcitabine resistance	Ovarian cancer	[187]
miR-195	Upregulated	NLRX1	Suppresses pyroptosis induced by enterovirus A71 in neuroblastoma cells	Neuroblastoma	[188]
miR-125b	Upregulated	FOXP3/caspase-1/GSDMD	Induces pyroptosis and restricts cell proliferation	Nasopharyngeal carcinoma	[189]
miR-22	Downregulated Downregulated Downregulated Downregulated	NLRP3 NLRP3 NLRP3 NLRP3/PI3K/AKT	Inhibits NLRP3 inflammasome activation, reducing melanoma cell activity Suppresses invasion, migration, and cell proliferation Suppresses invasion, migration, and cell proliferation Suppresses cell viability and EMT	Melanoma Colorectal cancer Oral squamous cell carcinoma Ovarian cancer	[190] [191] [192] [193]
WEE1	Upregulated	miR-138/SIRT1	Promotes metastasis	Glioma cancer	[194]
XRCC5	Upregulated	IGF2BP2/CLC3/SGK1	Aggravates tumor growth	Glioma cancer	[195]
PDIA3	Upregulated	miR-449a/XBP1	Induces chemoresistance, inhibits pyroptosis	Colorectal cancer	[196]
NEIL3	Upregulated	miR-1184/PIF1/AIM2	Regulates pyroptosis and influences radiotherapy efficacy	Lung adenocarcinoma	[197]
RNAPIBF1	Upregulated	Nrf2/EP300/SOD2	Correlated with poor prognosis	Lung adenocarcinoma	[198]
hsa_circ_0007312	Upregulated	miR-764/MAPK1	Inhibits pyroptosis, promotes osimertinib resistance	Lung adenocarcinoma	[199]
PUM1	Upregulated	UQCRC1/2	Inhibits pyroptosis, enhances the stability of mitochondrial complex III for ATP production in cancer cells	Esophageal squamous cell carcinoma	[200]

### Glioma

Glioma represents the most prevalent type of primary brain malignancy diagnosed, developing from neuroglial progenitor cells within the central nervous system (CNS). The standard care for glioma typically includes maximal safe resection, followed by adjuvant radiotherapy and chemotherapy with temozolomide (TMZ) [212, 213]. Nevertheless, the use of TMZ treatment in low-grade glioma can trigger hypermutation, leading to the progression of glioblastoma (GBM), identified as the most advanced stage of glioma, associated with a mere 6.8% five-year survival rate [214]. Therefore, the identification of new factors involved in the pathogenesis of gliomas, as well as the development of new therapeutic strategies, is being actively pursued [215–219]. Notably, recent research on the role of pyroptosis in glioma has offered promising horizons for devising more applicable therapeutic modalities.

#### Pyroptosis mechanisms and protein regulation

Zika virus (ZIKV) is an oncolytic virus that provides a novel platform for GBM cell death. However, keeping normal cells intact during Zika infection poses a major barrier to its application in cancer treatment. Kao et al. demonstrated that ZIKV requires GSDMD to induce efficient cell death. Notably, Zika protease cleaves GSDMD and produces IL-1β, whereas inhibition of GSDMD by a small-molecule abrogates Zika-induced cell death in both GBM and normal cells [220]. For clinical translation of Zika-associated therapy, several issues need to be addressed. First, patient‐to‐patient variability in GSDMD genotype may significantly affect susceptibility: variants that resist protease cleavage or fail to oligomerize blunt the effect. Thus, patient stratification would be essential [220]. Second, safety in the central nervous system is critical: activation of pyroptosis releases pro‐inflammatory cytokines (e.g., IL‐1β), which might damage healthy brain tissue or trigger deleterious neuroinflammation [220, 221]. Third, species specificity: most preclinical models are murine; but ZIKV protease cleavage of GSDMD appears human‐specific, so humanized or transgenic models will be needed to test efficacy and safety [220]. Finally, delivery, viral tropism, and control of viral spread must be resolved to ensure that the virus reaches tumor tissue while minimizing systemic dissemination or off‐target effects. With careful design (attenuation, targeting, regulated expression), the ZIKV–GSDMD axis holds promise, but moving toward clinical trials demands rigorous preclinical safety and patient selection data.

Deletion of AIM2 can result in markedly enhanced cell proliferation and resistance to TMZ treatment [222]. Guo et al. revealed that patients with upregulated caspase-6 tend to be more susceptible to TMZ, while patients with decreased caspase-6 respond better to immunotherapy [223]. Upregulation of guanine nucleotide-binding protein-4 (GNB4) in glioma cells increases cancer progression, while silencing GNB4 restrains tumor growth by promoting the expression of pyroptotic proteins and inflammatory factors via cyclic GMP–AMP Synthase – Stimulator of Interferon Genes (cGAS-STING) pathway [224]. Moreover, inhibition of Enhancer of Zeste Homolog 2 (EZH2) in GBM cell lines induces NLRP3/GSDMD-mediated pyroptosis. STAT3 serves as a downstream factor of EZH2, and their simultaneous inhibition leads to the expression of IL-1β and IL-18 [225]. Upregulation of NLRC4 in glioma patients contributes to poor prognosis and could serve as a diagnostic marker [40]. In C6 glioma cells, inhibition of NLRP3 through beta-hydroxybutyrate (BHB) reduces caspase-1 and suppresses cell migration, likely by reducing ACS oligomerization or preventing K^+^ efflux [226]. In vivo, MyD88 deficiency, a key adaptor protein in the activation of NF-κB pathway, restrains glioma development through p38 Mitogen-Activated Protein Kinase (p38-MAPK) signaling pathway [227]. Downregulating MyD88 mitigates LPS-induced cell pyroptosis and reduces the release of IL-1β and IL-18, suggesting a likely oncogenic role for MyD88 [228].

#### Non-coding RNA regulation

**Fig. 3:**
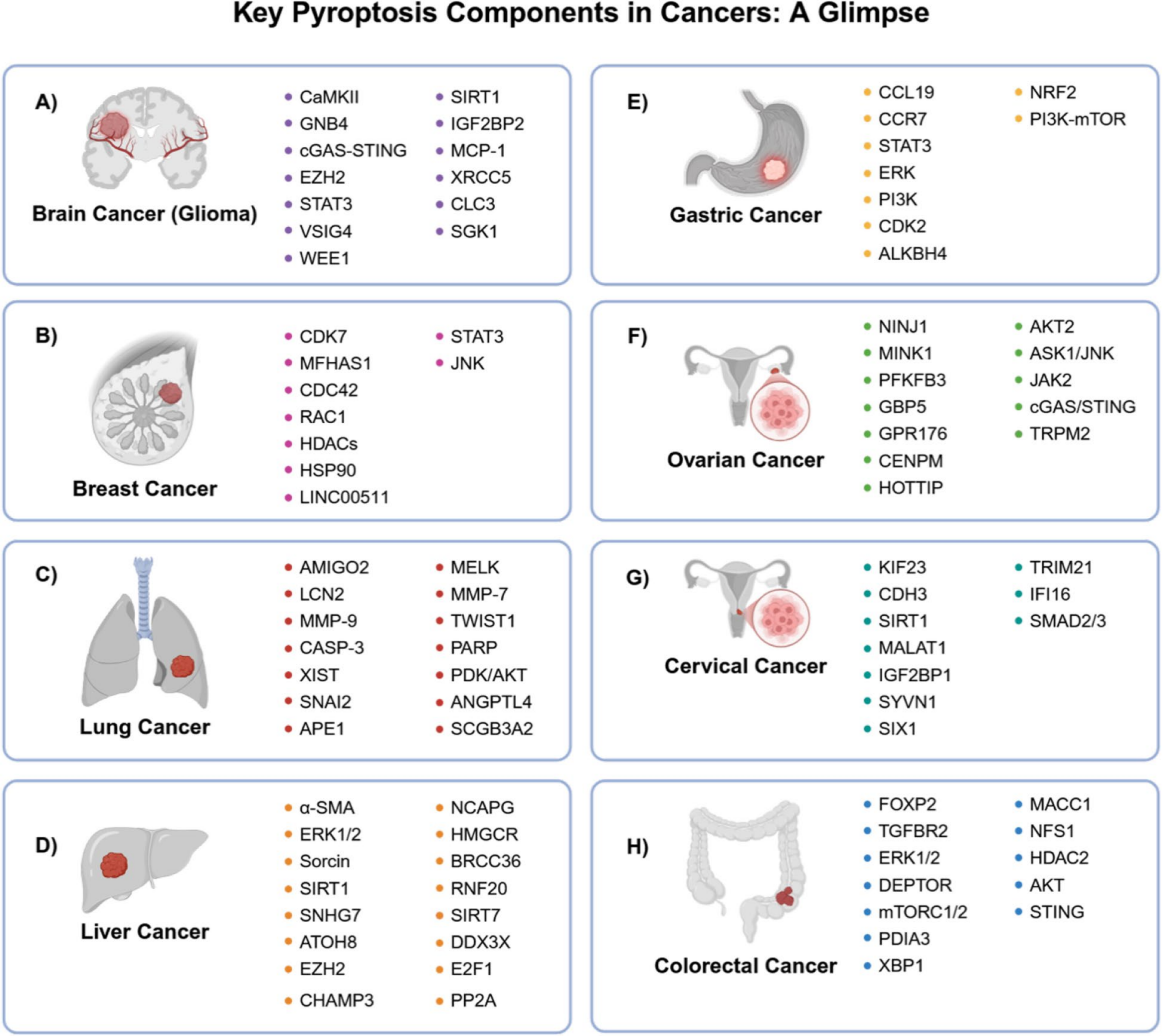
A diagram of the eight most common cancers and their altered genes associated with pyroptosis

Highlighting the crucial role of ncRNAs, Jiang et al. showed increased levels of caspase-1 expression in T98G and UB7 cells when miR-214 is downregulated (see Table 3 for a summary). Of note, the inhibition of miR-214 leads to a significant reduction in glioma cell proliferation and migration [172]. Elevated levels of circWEE1 enhance T98-G cell migration and invasion capabilities. Notably, circWEE1 expression, which is already elevated, increases even further in patients with either lymph node or distant metastasis compared to those with non-metastasis glioma. Mechanistically, circWEE1 negatively regulates miR-138, which in turn directly regulates sirtuin 1 (SIRT1) expression [194]. Additionally, upregulation of circXRCC5 is associated with glioma growth via the Chloride channel protein 3/Serum/Glucocorticoid-Regulated Kinase 1 (CLC3/SGK1) axis. CircXRCC5 exerts its effect by preserving the mRNA stability of CLC3 through interaction with IGF2BP2, which leads to accelerated expression of SGK1 mediated by PI3K/PDK1/AKT signaling [195]. Furthermore, miR-223-3p, a possible target of NLRP3, is downregulated in GBM cell lines, promoting cell proliferation and migration. Notably, treatment with a miR-223-3p mimic inhibits NLRP3 activation, reduces monocyte chemoattractant protein-1 (MCP-1), IL-1β, IL-8, and IL-18, thereby halting cell proliferation and migration [186]. Although various ncRNAs, such as miR-885-5P, miR-21/23a, and miR-195/205, have been linked to glioma and studied for their effects on apoptosis, proliferation, and migration, their potential involvement in crosstalk with pyroptosis requires further investigation [229–231]. Pyroptosis offers novel therapeutic strategies, such as targeting GSDMD, NLRP3, or ncRNAs like miR-214 and circWEE1, to enhance TMZ sensitivity, overcome resistance, and curb glioma progression (Fig. 3A).

### Lung cancer

Lung cancer is responsible for the most cancer-related fatalities and was the second most diagnosed cancer type in 2020 [232]. Approximately 80% to 85% of lung cancers are classified as non-small cell lung cancer (NSCLC), with its three main subtypes being adenocarcinoma, large cell carcinoma, and squamous cell carcinoma [233]. The remaining 15% of lung cancers are mostly classified as small cell lung cancer (SCLC), which represents an exceptionally aggressive phenotype with a 5-year survival of 7% [234].

#### Pyroptosis in lung cancer

To date, multiple studies have revealed the footprint of pyroptosis in varied subtypes of lung cancer. Gao et al. demonstrated an upregulation of GSDMD proteins in NSCLC compared to paired adjacent tumor tissues. Co-expression analyses revealed a relationship between EGFR/Akt signaling and GSDMD. Higher GSDMD levels were related to an adverse prognosis and cancer aggression (metastasis and larger tumor size). However, no association between increased GSDMD and poor prognosis is observed in squamous cell carcinoma. Of note, knockdown of GSDMD confined tumor growth both in vitro and in vivo. They explained that upon knockdown of GSDMD, NLRP3/caspase-1 signaling induced apoptosis instead of pyroptosis by recruiting caspase-3 and PARP [16]. In addition to GSDMD, Hung et al. reported that the expression levels of caspase-3, caspase-8, and GSDME is drastically increased in the majority of lung cancers compared to matched normal tissues. However, a positive link was observed between GSDME and postoperative survival outcomes [235]. Overexpression AIM2 in lung adenocarcinoma is thought to be related to a worse prognosis and induces immune escape by upregulating PD-L1 via the JAK-STAT3 pathway. Therefore, targeting AIM2 inhibition could be a promising strategy to stimulate the immune system [236, 237].

#### Non-Coding RNA regulation

Shi et al. demonstrated that miR-556-5p is significantly elevated in NSCLC resistant cisplatin. They found that knockdown of miR-556-5p could increase sensitivity to cisplatin by triggering NLRP3-mediated pyroptisis (see Table 3 for a summary) [185]. Similarly, overexpression of GSDME can promote cisplatin sensitivity and regress NSCLC by increasing the immune infiltration mediated by pyroptosis [48]. Consistent with this, adhesion molecule with IgG-like domain 2 (AMIGO2) protein attenuates cisplatin sensitivity by inhibiting GSDME-mediated pyroptosis. AMIGO2 is assumed to activate PDK1/AKT pathway, thus inhibiting the activation of caspase-8 and caspase-9/caspase-3 pathways [238]. Moreover, inhibition of Matrix Metalloproteinase-9 (MMP-9) by lipocalin 2 (LCN2) can reduce almonertinib resistance, suggesting that the LCN2/MMP-9 axis might offer a potential strategy for addressing drug resistance in lung adenocarcinoma [239]. Angiopoietin-like 4 (ANGPTL4) plays a role in glucose metabolism, and its upregulation induces gefitinib resistance by inhibiting NLRP3/ACS/caspase-8 pathway [240].

Recent studies have shown that the overexpression of lncRNA X inactive-specefic transcript (XIST) is linked to NSCLC. A study by Liu et al. revealed that knockdown of lncRNA XIST stimulates NLRP3 activation and affects ROS-induced pyroptosis through the alteration of miRNA-355/superoxide dismutase 2 (SOD2) pathway [153]. Secretoglobin family 3 A member 2 (SCGB3A2) is a small secretory protein mainly found in the Club cells of the airway epithelial and has several biological functions, including anti-cancer and anti-fibrotic features. In a study by Yokoyama et al., higher levels of SCGB3A2 were positively associated with lung adenocarcinoma survival outcomes. SCGB3A2 demonstrated significant anti-cancer activity against NSCLC human cell lines, but no effect was observed in SCLC human cell lines. They also found that SCGB3A2 and LPS induces syndecan 1 (SDC1), a cell surface receptor for SCGB3A2, and caspase-4, resulting in pyroptotic cell death [241].

#### Chemoresistance and therapeutic insights

Zhang et al. evaluated the relationship between the transcription factor P53 and pyroptosis in NSCLC for the first time. They reported a direct interaction between P53 and NLRP3, and that high P53 expression levels significantly inhibit cell growth [242]. Maternal embryonic leucine zipper kinase (MELK), a member of SNF1/AMPK family, is essential for mitotic progression. Lia et al. showed that high levels of MELK is negatively associated with lung adenocarcinoma survival. Knockdown of MELK results in reducing tumor cell invasion and migration, likely by diminishing the levels of matrix metalloproteinase 7 (MMP7), N-catenin, twist family BHLH transcription factor 1 (Twist1), and snail family transcriptional repressor 2 (Snai2). Taken together, pyroptosis could be a key contributor to future lung cancer treatment protocols (Fig. 3C).

### Breast cancer

Breast cancer is the most prevalent cancer in woman with over 2 million new cases being affected in 2020 worldwide [243].

#### Pyroptosis activation and mechanisms

Pyroptosis can be activated by PD-L1, TNF-α, chemotherapy or antibiotics. Hou et al. demonstrated that PD-L1 is capable of transforming TNF-α-induced apoptosis to pyroptosis, suggesting a non-immune checkpoint activity of PD-L1. Mechanistically, phosphorylated STAT3 can directly bind to nuclear-translocated PD-L1, and this complex interacts with the GSDMC promotor. In MDA-MB-231 breast cancer cells, high expression of GSDMC/caspase-8 via TNF-α inducing, is correlated with poor survival [55]. Another study showed the expression levels of GSDMC in breast cancer cells are significantly upregulated compared to normal breast cells (MCF10A) [244]. LINC00511/has-miR-573 regulatory pathway has also shown to mediate pyroptosis through GSDMC upregulation and is related to tumor immune infiltration and dismal prognosis (see Table 3 for a summary) [163]. The expression analysis of caspase-1, GSDMD, and IL-1β in 108 cases of breast cancer by means of immunohistochemistry method demonstrated that the components are positively correlated with each other. Moreover, their higher expression levels are associated with lower likely of lymph node metastasis, histopathological grade, clinical stage, and smaller tumor size as well [245].

#### Therapeutic interventions

Yang et al. explored the effects of histone deacetylase inhibitors (HDACi), a new class of anti-cancer agents, in triple negative breast cancer (TNBC). HDACi could markedly decrease cell proliferation, and induce pyroptotic cell death. Notably, TNBC cells displays the highest amounts of GSDME, with GSDMA, GSDMB, and GSDME levels increasing further after HDACi treatment. In vivo experiments showed that HDACi can increase granzyme B in tumors, diminish tumor growth, and promote CD8^+^ lymphocyte and CD11b^+^ cell infiltration [246].

#### Role of GSDME isoforms

In terms of splicing isoforms and their effect on breast cancer cells’ behavior, Hergueta-Redondo et al. showed that among four identified GSDMB isoforms, GSDMB-1 and GSDMB-2 isoforms promote invasion and motility in MCF-7 cell line, whereas the suppression of these isoforms in the HCC1954 cell line decrease the invasive and migratory phenotype. Interestingly, although both isoforms engage with the chaperone heat shock protein 90 (Hsp90), increase invasion and motility through activation of Rho-GTPase Ras-related C3 botulinum toxin substrate 1 (Rac-1) and Cell division control protein 42 (Cdc-42) in vitro, only GSDMB-2 relies on Hsp90 for its stability, and promotes metastasis and tumor progression in xenograft mouse models [247].

#### Epigenetic regulation

Epigenetic alterations of GSDME, particularly methylation have been reported by several studies [65, 248, 249]. Kim et al. showed that the methylated GSDME is associated with lymph node metastasis in patients and the methylation status can be restored by 5-aza-dC treatment. In addition, knockdown of GSDME revealed promoted cellular invasion [248]. Dysregulation of cyclin-dependent kinase 7 (CDK7) is a common feature of many tumors, and CDK7 inhibition has been shown to repress colony formation and cell proliferation in breast cancer by elevating expression levels of P53 and GSDME [250].

### Gastric cancer

Gastric cancer (GC) is among the top five most frequently diagnosed cancers globally and stands as the fourth main contributor to cancer-related mortality worldwide [251]. Chronic gastric mucosa infection expedites the gradual progression of atrophic gastritis and intestinal metaplasia, thereby increasing the growth of GC. Moreover, GC shows considerable molecular and phenotypic heterogeneity. The standard approach for advanced GC typically involves a multi-line approach to chemotherapy, starting with a combination of platinum and fluoropyrimidine agents. Nevertheless, despite these interventions, the median survival remains less than one year [252].

#### Pyroptosis and molecular pathways

Wang et al. revealed that GSDMD inhibits the activation of the STAT3, ERK, and PI3K pathways, resulting in the disruption of CyclinA2/CDK2 function. This, in turn, leads to cell cycle arrest and a notable decrease in the proliferation of GC cells [15]. Under hypoxic conditions, Hypoxia-Inducible Factor 1-α (HIF-1α) can induce mitochondrial dysfunction and the release of ROS, activating NLRP3/caspase-1/GSDMD pathway and thereby aggravating the development of GC. Dynamin-related protein 1 (DRP1) is a cytoplasmic GTPase involved in mitochondrial fission, and its N6-Methyladenosine (m6A) methylation, which is induced by upregulation of the methyltransferase-like 3/insulin-like growth factor 2 mRNA binding protein 3 (METTL3/IGF2BP3) axis is responsible for promoting ROS production [253]. Additionally, CagA, an important effector protein of *Helicobacter pylori*, promotes GC migration and invasion by activating NLRP3, while the inhibition of ROS by N-Acetylcysteine (NAC) can block NLRP3 activation [254]. C–C Motif Chemokine Ligand 19 (CCL19) is a chemokine, and its upregulation inhibits GC cell proliferation and tumor growth by upregulating the C–C Motif Chemokine Receptor 7 (CCR7)/AIM2 axis, while silencing AIM2 abrogates the effects of CCL19 on tumor growth [255].

#### Non-coding RNA regulation

In terms of the regulatory role of ncRNAs, miR-125a is a tumor suppressor whose diminished levels are associated with unfavorable prognosis in GC (see Table 3 for a summary) [256, 257]. Ren et al. reported a downregulation of lncRNA ADAMTS9-AS2 and an upregulation of miR-223-3p in GC tissues compared to the normal tissues. Of note, patients with elevated levels of lncRNA ADAMTS9-AS2 and reduced miR-223-3p tend to experience more favorable prognoses, along with lower proliferation and mortality. Additionally, it was found that lncRNA ADAMTS9-AS2 triggers the NLRP3 in GC cells via miR-223-3p downregulation [154].

#### Chemoresistance and therapeutic insights

Chemoresistance is a major challenge in achieving successful cancer treatment, and various potential solutions have been proposed to address this barrier [258–260]. In terms of chemoresistance regulation, Li et al. provided evidence that PD-L1 negatively regulates NLRP3 in patients with cisplatin-resistant cells. Therefore, the crosstalk between PD-L1 and NLRP3 to activate pyroptosis, may be important in addressing these resistance challenges [261]. Furthermore, caspase-3/GSDME plays a dual role in regulating pyroptosis and apoptosis. The use of CRISPR-Cas9 technology to knock out GSDME, following treatment with 5-fluorouracil (5-FU), causes a shift from pyroptosis to apoptosis, mediated by caspase-3 [262]. It has been shown that upregulation of AlkB Homolog 4 (ALKBH4) inhibits trimethylation of lysine 4 on histone (H3K4me3) modification, thereby leading to GSDME suppression and reduced 5-FU sensitivity [263]. Nuclear factor erythroid 2-related factor 2 (NRF2), known as a nuclear transcription factor, plays a crucial role in HER2 resistance through its activation of the PI3K-mTOR signaling pathways [264, 265]. While the primary role of NFR2 in GC pyroptosis has remained unidentified, we speculate that it indirectly contributes to pyroptosis by means of NF-κB, NLRP3, and PI3K/AKT/NFR2 signaling axes [266] (Fig. 3B).

### Colorectal cancer

Colorectal cancer (CRC) is a significant global health issue, accounting for the third highest number of cancer diagnoses and the second highest number of cancer-related deaths globally [267, 268]. CRC is a multifactorial disease influenced by lifestyle, ethnicity, age, and dysregulated signaling pathways, such as proto-oncogene activation and tumor suppressor inactivation [269, 270].

#### Pyroptosis and molecular pathway

Regarding the role of pyroptosis in CRC, Liao et al. found that forkhead box P2 (FOXP2) is downregulated in both colitis and tumor tissues, which is associated with poor survival outcomes. The loss of FOXP2 inhibits pyroptosis through the caspase-1/GSDMD pathway while increasing the levels of proliferation-associated proteins such as proliferating cell nuclear antigen (PCNA) and cyclin D1 [271]. Additionally, pyroptosis is regulated post-transcriptionally by miR-21-5p through its effects on TGF-β1 (see Table 3 for a summary) [179]. Miguchi et al. demonstrated that knockout of TGF-β receptor-2 (TGFBR2) and APC results in the upregulation of GSDMC and enhanced cell proliferation. Conversely, silencing GSDMC caused a notable decline in cell proliferation and tumorigenic potential in CRC cell lines [272]. Moreover, GSDME encourages tumor cell proliferation and PCNA expression by enhancing HMGB1 release through the Extracellular Signal-Regulated Kinase 1/2 (ERK1/2) pathway [273]. In contrast, Guo et al. found that GSDME suppresses cell proliferation and tumor growth and prevents cell cycle progression by inhibiting mechanistic target of rapamycin complex 1/2 (mTORC1/2) signaling pathways [274]. It has been shown that the upregulation of lncRNA RP1-85F18.6 correlates with the reduction of pyroptosis, leading to cell cycle disruption and enhanced cell proliferation and invasion [155]. Dysregulation of AIM2 promotes uncontrolled tumors growth by affecting Akt phosphorylation. In cells lacking AIM2, Akt activation mediated by DNA-dependent protein kinase (DNA-PK) reduces tumor burden. Therefore, Akt inhibitors could be rational drug design targets for AIM2-deficent cancers [275].

#### Chemoresistance and therapeutic implications

To address chemoresistance in CRC, Lin et al. demonstrated that the circular RNA Protein Disulfide Isomerase A3 (circPDIA3) induces chemoresistance by curbing pyroptosis through a positive feedback loop involving miR-449a/X-Box Binding Protein 1 (XBP1). Mechanistically, circPDIA3 interacts with the C-terminal of GSDME, enhancing its autoinhibitory effects by inhibiting GSDME palmitoylation mediated by Zinc Finger DHHC-Type Palmitoyltransferase 3/17 (ZDHHC3/17) [196]. Another inducer of chemoresistance is *Fusobacterium nucleatum*, which inhibits the caspase-3/GSDME axis through the Hippo signaling pathway [276]. In addition, CRC cells with higher GSDMB expression levels have been shown to be more responsive to 5-FU treatment and are associated with elevated CD68 or S100 calcium binding protein A8 (S100A8) immune cell profiles [277]. Metastasis-associated in colon cancer 1 (MACC1) is an oncogene whose upregulation inhibits the cleavage of GSDME, leading to resistance to irinotecan [278]. Inhibition of cysteine desulfurize (NFS1), which is transcriptionally regulated by the myelocytomatosis oncogene (MYC) has been identified as a viable strategy to resolve chemoresistance to oxaliplatin [279]. Epigenetic downregulation of NLRP3/GSDMD by histone deacetylase 2 (HDAC2) can reduce anti-tumor therapeutic efficacy. HDAC2 exerts its inhibitory effect by hindering the formation of the H3K27ac/BRD4/p-P65 complex [280].

#### Microbiota and signaling pathways

A recent study provided evidence that gut microbiota can activate GSDMD via the NLRP3-mediated pathway. Intriguingly, the activation of GSDMD led to the development of CRC, while its inactivation was linked to an increased number of macrophages and reduced infiltration of immature myeloid cells, suggesting the potential use of NLRP3 inhibitors to counteract tumorigenesis [281]. Another pathway that contributes to GSDMD-mediated pyroptosis, which may help attenuate tumorigenesis, adhesion, and invasion in CRC, involves the upregulation of the interferon gene stimulator (STING)-mediated spleen tyrosine kinase (Syk) signaling pathway. Activated Syk plays a key part in upregulation of the type 1 IFN-related TANK-Binding Kinase 1 (TBK1)- Interferon Regulatory Factor 3 (IRF3) pathway [282]. Altogether, the dual role of pyroptosis in CRC ranging from tumorigenesis to tumor suppression demonstrates its double-edged nature, necessitating continued research to uncover more unknowns (Fig. 3H).

### Liver cancer

Liver cancer is responsible for the third-highest number of cancer-related deaths worldwide and is the sixth most frequently diagnosed malignancy [283]. The vast majority of liver cancer cases are due to hepatocellular carcinoma (HCC), which represents about 75% of all primary liver cancers [284].

#### Pyroptosis mechanisms and protein regulation

NEK7 is crucial in regulating of NLRP3 activation and is correlated with GSDMD in HCC. Knockdown of NEK7 impairs the activation of hepatic satellite cells, cancer-stromal interaction, and the ability of cells to migrate and invade, potentially by decreasing α -smooth muscle actin (α-SMA) and p-ERK1/2 [285]. In addition, Soluble resistance-related calcium-binding protein (Sorcin) is upregulated in HCC tissues and cell lines, while components of pyroptosis are downregulated. Research has shown that Sorcin negatively interacts with NLRP3; thus, silencing Sorcin activates pyroptosis, which leads to a reduction in cell growth, invasion, and migration. Notably, caspase-1 inhibition could partially restore tumor growth, which is otherwise impeded by Sorcin knockdown [286]. Moreover, SIRT1 is upregulated in HCC and represents a negative correlation with the N-terminal of GSDME. Silencing SIRT1 aggravates GSDME-mediated pyroptosis through mitochondrial damage. However, when SIRT1 is silenced, inhibiting GSDME reduces pyroptosis and shifts it towards apoptosis [287]. Chen et al. demonstrated that knockdown of GSDME remarkably suppresses tumor progression in HCC [288]. Another study reported that upregulation of GSDMC in HCC contributes to cell proliferation and tumor progression [87].

#### Non-coding RNA and viral influences

LncRNA small nuclear RNA host gene 7 (SNHG7) is upregulated and negatively associated with the activation of NLRP3 in HepG2 and SK-hep-1 cells (see Table 3 for a summary). Additionally, SNHG7 is thought to function as a competing endogenous RNA of miR-34a, with SIRT1 shown to be directly targeted by miR-34a. knockdown of SNHG7 decreases SIRT1 expression levels while promoting NLRP3/caspase-1/IL-1β pyroptosis components through a regulatory loop comprising lncRNA SNHG7/miR-34a/SIRT1 (see Table 3 for a summary) [156]. It has been shown that hepatitis C virus can induce both apoptosis and pyroptosis in infected cells. While the infected cells undergo pyroptosis, bystander cells typically undergo apoptosis and remain unaffected by pyroptosis [289]. Additionally, overexpression of atonal BHLH transcription factor 8 (ATOH8) is associated with immune escape of hepatitis B virus through the downregulation of pyroptosis components [290]. Hepatitis B virus X protein (HB_X_) noticeably suppresses AIM2 by increasing the stability of Enhancer of zeste homolog 2 (EZH2). Ubiquitination of AIM2 by HB_X_ leads to cell migration, metastasis, formation of cell pseudopodium, and epithelia-mesenchymal transition, while reintroduction of AIM2 reverses these effects. Fibronectin 1 is a downstream target of AIM2, with its levels being inversely regulated by AIM2. Silencing fibronectin 1 markedly impedes cell migration induced by AIM2 abolition [291]. Another study revealed that downregulation of AIM2 in HCC is associated with increased cell proliferation and colony formation via activation of the mTOR/S6K1 pathway [292]. Wang et al. found that 3-hydroxy-3-methyl-glutaryl-coenzyme A reductase (HMGCR) is localized in the mitochondria but is translocated to the endoplasmic reticulum during pyroptosis. In addition, BRCA1/BRCA2-containing complex 3 (BRCC36) deubiquitinates HMGCR, thereby promoting pyroptosis [293].

#### Therapeutic and chemoresistance insights

Research has shown a two-step activation process in TLR4-mediated pyroptosis induced by geranylgeranoic acid. First, caspase-4 is activated, leading to the translocation of GSDMD to plasma membrane. This is followed by a gradual second increase in intracellular Ca^2+^ levels, which leads to caspase-1 cleavage through NF-κB/NLRP3 activation [294]. 17β-estradiol exerts its inhibitory effect on HCC cells by inducing NLRP3/caspase-1 pyroptosis pathway. In cells treated with 17β-estradiol, adding 3-methyladenin to the medium aggravates pyroptosis activation [295]. Similarly, a study by Awwad et al. supports the anti-cancer effects of 17β-estradiol on HCC cells through pyroptosis [296]. Overexpression of ring finger protein 20 (RNF20) in liver cancer is positively associated with postoperative survival rates and inhibits cell proliferation and metastasis by promoting NLRP3 ubiquitination [297]. Regarding drug resistance in HCC, inhibition of SIRT7 leads to depletion of dead-box helicase 3 X-liked (DDX3X), which can reverse acquired sorafenib resistance by hampering NLRP3 assembly. This disruption ultimately suppresses overactive ERK1/2 signaling by reducing IL-1β mediated by NLRP3 [298]. A recent study found that E2 promoter binding factor 1 (E2F1) acts as the upstream activator of Non-SMC Condensin I complex subunit G (NCAPG). Knockdown of NCAPG activates pyroptosis in HCC cell lines through the E2F1/NCAPG/NLRP3 regulatory mechanism [299]. HGS-ETR1/2 is a monoclonal antibody that interacts with death receptor4/5 (DR4/5) and is capable of inducing GSDME-mediated pyroptosis. Mechanistically, suppression of the AKT pathway or silencing carboxypeptidase A4 (CPA4) both encourage pyroptosis, whereas CPA4 overexpression prevents it. CPA4 regulates AKT phosphorylation through protein phosphatase 2 (PP2A), leading to reduced CPA4 expression due to AKT inhibition, thus creating a positive feedback loop [300] (Fig. 3D).

### Cervical cancer

Cervical cancer (CC) is the fourth most frequently occurring cancer in women across the globe [301], and ranks second among women aged 15–44 years old [302]. CC is primarily caused by persistent infections with high-risk types of human papillomavirus (HPV) [303]. In 2020, an estimated 604,127 cases with 341,831 deaths due to CC were reported globally [304].

#### Pyroptosis mechanisms and protein regulation

In regard to the role of pyroptosis in CC, Liu et al. found that downregulation of kinesin family number 23 (KIF23) reduces proliferation, invasion, and migration of CC cells, while its upregulation promotes tumorigenic phenotype, likely through the inhibition of NLRP3-mediated pyroptosis [305]. Crosstalk analyses of pyroptosis subtypes confirm that knockdown of Cadherin-3 (CDH3) inhibits CC cell proliferation, highlighting its role in cell growth regulation [306]. In addition, overexpressed SIRT1 supports the growth of HPV-infected CC cells by suppressing AIM2 activity [307].

#### Non-coding RNA regulation

The overexpression of lncRNA Metastasis Associated Lung Adenocarcinoma Transcript 1 (MALAT1) is believed to block pyroptosis through the miR-214/SIRT1 regulatory axis (see Table 3 for a summary) [157]. Moreover, upregulated lncRNA Leucine Rich Repeat Containing 75 A Antisense RNA 1 (LRRC75A-AS1) in tumor tissues plays a role in epithelial-mesenchymal transition through the activation of IL-1β/mothers against decapentaplegic homolog 2/3 (smad2/3) signaling. Mechanistically, LRRC75A-AS1 competitively interacts with Insulin-like growth factor 2 mRNA-binding protein 1 (IGF2BP1) to destabilize E3 ubiquitin-protein ligase synoviolin (SYVN1) mRNA, leading to SNVN1-mediated NLRP3 ubiquitination degradation and subsequent activation of the IL-1β/smad2/3 signaling [158]. In another pathway, LRRC75A-AS1 delivered by M2 macrophage exosomes has been shown to promote cancer progression by inhibiting miR-429, which upregulates sine oculis homeobox homolog 1 (SIX1) levels and activates the STAT3/MMP-9 axis [308]. Yu et al. indicated that miR-214 is downregulated in both CC patients and cell lines. However, overexpression of miR-214 can induce NLRP3-mediated pyroptosis, thereby promoting cell death [94].

#### Therapeutic opportunities

The HPV oncoprotein E7, a critical regulator of immune surveillance, restrains pyroptosis and IL-1β/−18 production by interacting with IFN-γ-inducible protein 16 (IFI16) and tripartite motif-containing protein 21 (TRIM21). Mechanistically, HPV E7 triggers the E3 ligase TRIM21 to target the IFI16 inflammasome for degradation [309]. Additionally, the viral oncoprotein E6 exerts its inhibitory effect on IL-1β production by abrogating the interactions between P53 and Interferon Regulatory Factor 6 (IRF6) [310]. In HeLa cells infected with *Chlamydia trachomatis*, the secretion of virulence proteins triggers K^+^ efflux through glibenclamide-sensitive channels. This leads to ROS production and subsequent NLRP3-dependent caspase-1 activation, which is crucial for intracellular growth of *Chlamydia* [311]. Highlighting crosstalk between the immune system and GSDM family, granzyme B released from immune cells can induce GSDME cleavage in HeLa cells [138]. Taken together, we emphasized the indispensable roles of pyroptosis in CC identified to date and highlighted the novel opportunities it presents for addressing the disease (Fig. 3G).

### Ovarian cancer

Ovarian cancer (OC) is one of the leading causes of mortality among women, ranking as the eighth most diagnosed and deadliest malignancy globally. In 2020, approximately 314,000 women were affected by OC, and over 207,000 died from it [284, 312]. Studying the impact of pyroptosis in the context of OC could provide valuable insights to overcome current challenges and help devise rational therapeutic approaches.

#### Pyroptosis mechanisms and protein regulation

Ninjurin 1 (NINJ1) contributes to the disruption of the plasma membrane, and its expression levels decrease from early to late stages in serous OC and are associated with poorer overall survival [313]. Research has shown that NINJ1 is downstream of GSDMD [314]. However, a study by Berkel and Cacan found that higher levels of GSDMD are associated with decreased levels of NINJ1 in tumor tissues compered to adjacent stromal cells, which represents a different finding from previous studies [313]. Calbay et al. uncovered that treatment of OC cells with docosahexaenoic acid markedly promotes caspase-1 abundance and activation, intriguingly with ASC being dispensable for this process. The synthesis of caspase-1 is thought to be mediated by P38^MAPK^/MAPK-interacting serine/threonine-protein kinase 1 (MNK1) signaling pathway [315]. As a key regulator of glycolysis, upregulation of 6-phosphofructo-2-kinase/fructose-2,6-biphosphatase 3 (PFKFB3) promotes the Warburg effect, cell proliferation, and metastasis. In addition, PFKFB3 suppresses NLRP3 and caspase-3/−9, suggesting an inhibitory role of PFKFB3 in pyroptosis [316]. Silencing of NLRP3 hinders cell proliferation, migration, and invasion, and sensitizes cisplatin-resistant OC A2780 and SKOV3 cell lines. Additionally, inhibition of NLRP3 blocks epithelial-mesenchymal transition by promoting E-cadherin and reducing N-cadherin, vimentin, and fibronectin [317].

#### Non-coding RNA regulation

Concerning the crucial regulatory role of ncRNAs, lncRNA HOXA Transcript at the Distal Tip (HOTTIP) is upregulated in OC tissues and cell lines, and silencing HOTTIP inhibits cell proliferation and NLRP1-mediated pyroptosis (see Table 3 for a summary). Mechanistically, HOTTIP negatively regulates miR-148a-3p, leading to increased AKT serine/threonine kinase 2 (AKT2) expression levels and subsequent inhibition of Apoptosis Signal-regulating Kinase 1 (ASK1)/JNK signaling [159]. Moreover, miR-22 interacts negatively with NLRP3. miR-22 impairs cell viability and epithelial-mesenchymal transition by targeting NLRP3 and inhibiting PI3K/AKT signaling [193]. Upregulation of miR-624-5p can increase NLRP3 expression, which promotes resistance to gemcitabine through the activation of IL-1β, epithelial-mesenchymal transition, and Wnt/β-catenin signaling pathways [187].

#### Chemoresistance and tumor microenvironment

Higher levels of GSDMD in omental adipocytes are linked to a worse prognosis and chemoresistance in advanced-stage OC. GSDMD-mediated pyroptosis in omental adipocytes leads to the release of free fatty acids and ATP, resulting in macrophage infiltration and uptake of these released factors by OC cells to increase chemoresistance [318]. A recent study revealed that guanylate binding protein 5 (GBP5) can induce caspase-1/GSDMD-dependent pyroptosis through the JAK2/STAT1 pathway and serve as an immunoreactive modulator through the overexpression of CXCL9/10/11, as well as M1 macrophage infiltration/polarization [319]. Knockdown of centromere protein M (CENPM) impedes proliferation, invasion, and migration of OC cells by triggering NLRP3/caspase-1/GSDMD axis and activating cGAS-STING pathway [320]. Treatment of high-grade serous ovarian carcinoma with progesterone significantly inhibits precancerous cell viability through the IL-6/ROS/NLRP3/GSDMD pathway [321]. An in-silico study revealed that Transient Receptor Potential Melastatin 2 (TRPM2) expression positively correlates with NLRP3, NLRC4, NOD1/2, and IL-1β [322]. However, further validation is needed to determine whether TRPM2 is involved in pyroptosis (Fig. 3F).

### Cross-cancer patterns and perspectives

Up to this point, we have reviewed pyroptosis components, including gasdermins, caspases, and inflammasomes within individual cancer types. While informative, this approach is largely descriptive and does not reveal overarching trends or translational insights. In this section, we shift focus to a cross-cancer perspective, aiming to synthesize patterns across tumor types, highlight recurring pro- or anti-tumor behaviors, identify context-dependent discrepancies, and propose hypotheses for therapeutic targeting. By integrating findings across cancers, we provide a framework to guide both mechanistic studies and potential clinical applications of pyroptosis modulation (Table 2).

Among the pyroptosis-related targets, caspase-3 and caspase-8 require particularly cautious consideration as therapeutic targets, since their activation can yield either anti- or pro-tumor outcomes depending on the downstream pathways engaged. For instance, caspase-3 activation in ovarian cancer following VP-16 treatment can paradoxically drive tumor repopulation through iPLA2–PGE2–FAK signaling, whereas in combination with proteasome inhibitors it induces robust apoptosis [100, 101]. Likewise, in glioblastoma, caspase-8 is often co-opted to promote NF-κB–driven cytokine production, angiogenesis, and migration instead of cell death [41]. These examples illustrate that caspase activity is not intrinsically pro- or anti-tumor but context-dependent. From a translational standpoint, this suggests that therapeutic strategies should avoid directly targeting caspase activation and instead intervene at downstream effectors, such as blocking PGE2/FAK or NF-κB pathways to redirect signaling toward anti-tumor outcomes while minimizing pro-tumor escape routes.

One important but often overlooked challenge is that the role of caspase signaling may be stage-dependent rather than uniform across tumor evolution. The central problem is that a molecule can act as a tumor suppressor in early disease yet switch to a tumor-promoting role once the tumor is established. A clear example comes from hepatocellular carcinoma, where caspase-8 supports apoptotic clearance and suppresses initiation in early stages, but in advanced tumors its activation fuels compensatory proliferation and microenvironmental remodeling that accelerate progression [82]. This duality highlights why therapeutic interventions that ignore tumor stage may risk backfiring. Clinically, incorporating stage as a key stratification factor, together with molecular and microenvironmental features, could improve predictive models and guide the timing of caspase-targeted strategies, ensuring that interventions are applied when they are most likely to generate durable anti-tumor benefit.

Analysis of the patterns summarized in Table 2 suggests that among the cancers examined, gastric cancer and cervical cancer display comparatively more consistent anti-tumor functions of pyroptosis components. This relative consistency positions gastric and cervical cancers as promising models for translational strategies targeting pyroptosis. In terms of patterns among inflammasome components, NLRP3 predominantly exhibits pro-tumor effects across multiple cancers, whereas AIM2 largely shows anti-tumor activity. Data on NLRC4 are limited, preventing consistent conclusions at this stage. Of note, GSDME generally acts as a robust anti-tumor effector across cancers (Table 2), except for liver and glioblastoma cancers. In glioblastoma, GSDME paradoxically promotes tumor progression: although cleavage occurs, active membrane repair mechanisms prevent effective pyroptosis, while GSDME still facilitates tumor cell invasion and suppresses T cell infiltration [42]. These observations provide a mechanistic rationale for its pro-tumor behavior in glioblastoma, while reinforcing that in other cancers, precise modulation of GSDME via epigenetic activation or inhibition remains a promising therapeutic strategy. It should be noted that future studies must incorporate tumor stage, cell type specificity, and therapy resistance profiles into their design, as these variables critically shape whether pyroptosis acts as a friend or foe in the TME.

These findings highlight several mechanistically grounded, testable hypotheses for future exploration. First, quantitative thresholds of pyroptosis component expression, such as caspase-3/8, GSDME, and NLRP3 may determine whether the net effect is anti- or pro-tumor, suggesting that fine-tuned modulation rather than binary activation/inhibition could optimize therapeutic impact. Second, spatiotemporal context within the tumor microenvironment, including immune cell composition, cytokine gradients, and metabolic state likely dictates the balance between tumor suppression and inadvertent pro-tumor inflammation, emphasizing the need for precise, stage- and cell type-specific interventions. Third, combinatorial approaches integrating epigenetic modulators, non-coding RNAs, or nanoparticle-based delivery systems could enable selective activation or repression of pyroptosis pathways, providing a controllable framework to leverage pyroptosis for therapy while minimizing adverse effects. Collectively, addressing these open questions offers a rational, data-driven roadmap for designing next-generation interventions.

#### Patterns and therapeutic potential of ncRNA-mediated pyroptosis regulation

This subsection synthesizes cross-cancer patterns of ncRNA-mediated pyroptosis regulation, focusing on recurring targets, regulatory axes, and context-dependent effects (Table 3). Rather than cataloging individual reports, we analyze common mechanistic themes and highlight ncRNAs with potential therapeutic relevance, setting the stage for subsequent discussion of nanoparticle-based strategies for precise modulation.

Across cancers, NLRP3, GSDMD, and SIRT1 emerge as the most recurrent ncRNA-regulated pyroptosis components. Analysis of Table 3 reveals three dominant regulatory mechanisms: (1) lncRNAs as miRNA sponges, preventing miRNAs from repressing targets such as NLRP3 or GSDMD; (2) miRNAs directly targeting mRNAs to suppress SIRT1 or inflammasome components; and (3) circular RNAs modulating mRNA stability or engaging RNA-binding proteins to fine-tune expression.

It should be noted that direct targeting of NLRP3 carries inherent risks due to its central role in multiple signaling networks, which may lead to off-target effects and unpredictable cellular responses. A more precise strategy is to leverage endogenous ncRNAs that naturally regulate NLRP3 activity through key intermediates such as SIRT1. Notably, regulatory axes like SNHG7/miR-34a/SIRT1 and MALAT1/miR-124/SIRT1, which converge on SIRT1 and NLRP3 indirectly, could yield synergistic enhancement of pyroptotic responses, particularly in drug-resistant tumors, while minimizing systemic toxicity [156, 157]. Translationally, targeted delivery via nanoparticles offers a promising avenue to implement these strategies in combination with conventional chemotherapy, enabling precise, context-specific activation of pyroptosis in cancer cells. This is consistent with rapidly expanding applications of nanotechnology in oncology for enhancing drug delivery, drug safety and diagnosis of various cancer types [323–327].

Despite their potential, ncRNA-based strategies to modulate pyroptosis face practical translational hurdles. ncRNA instability, rapid degradation in circulation, and off-target interactions complicate systemic delivery, while tumor heterogeneity and TME factors, such as immune infiltration and stromal composition dictate context-specific responses [328]. To overcome these barriers, nanoparticle-mediated delivery can enhance tumor-specific targeting, and simultaneous modulation of synergistic ncRNA axes (e.g., SIRT1–NLRP3-related pathways) may amplify therapeutic effects. Importantly, patient stratification based on tumor stage, immune context, and resistance profile is essential to maximize efficacy and minimize pro-inflammatory or pro-tumor consequences.

## Remodeling the tumor immune microenvironment

The role of pyroptosis in the modulation of TME is exemplified as two sides of the same coin in terms of tumorigenesis and antitumor immunity. On the one side, pyroptosis can induce tumor progression, invasion, metastasis, and transition of normal cells into cancerous cells via establishing long-term chronic inflammation. On the other side, pyroptosis behaves as a tumor suppressor by means of activating inflammasomes and release of cytokines to induce acute inflammation. Uncovering the hidden and complicated pattern of immune regulation derived by pyroptosis-related pathways is of paramount importance to hinder chronic inflammation and encourage tumor suppression.

### TME components and metabolic alterations

The TME comprises key elements like myeloid-derived suppressor cells (MDSCs), which suppress T-cell activity, and tumor-associated macrophages (TAMs), which are simply classified as M1 macrophage (proinflammatory phenotype) and M2 (anti-inflammatory phenotype), and promote tumor growth and immune evasion. Additionally, cancer-associated fibroblasts (CAFs) reshape the extracellular matrix (ECM), promoting tumor cell proliferation and invasion. Adipocytes and mesenchymal stem cells (MSCs) also contribute to the metabolic and structural remodeling of the TME. Metabolic alterations disrupt hemostasis, predisposing the tumor to proliferation and metastasis by establishing a low PH (PH < 4) and hypoxia, which restrict the functions of CTLs [329]. In a hypoxic condition, TNF-α upregulates GSDMC expression through caspase-8 activation [55]. Upregulation of hypoxia-inducing factor 1α (HIF-1α) promotes the production of NLRP3/caspase-1/GSDMD, GSDME and cytokines like TNF-α, IL-1β/−10, thereby aggravating cell proliferation and invasion in glial cells [330]. Low glucose and high cholesterol levels lead to T cell exhaustion, weakening the immune response. While CD8^+^ memory T cells can activate AKT-driven glycolysis through T cell receptor and CD28 signaling to produce IFN-γ, the lack of glucose limits their ability to function effectively, enabling tumors to escape the immune system [331]. M2 phenotype of TAMs release TGF-β and arginase 1, that not only attenuate the function of NK cells and CD8^+^ T cells but also have cross talk with Th1 and regulatory T cells (Treg) through the secretion of IL-10 and TGF-β [332].

### Pyroptosis pathways and immune regulation

It has been shown that the activation NLRP3 through HMGB1 promotes acute myeloid leukemia [333]. HMGB1 enhances the release of IL-10 from MDSCs, promoting crosstalk between macrophages and MDSCs L-selection on T cell, which disrupts their migration to lymph nodes [334]. Besides HMGB1, overexpression of exosomal HMGB3 is associated with poor overall survival, facilitating M2 macrophage polarization by triggering NLRP3, while knockdown of HMGB3 could restrain both NLRP3 and M2 macrophage activation [335]. NLRP3 activation is linked to the infiltration of MDSCs and TAMs in breast cancer [336]. Research indicates that LPS-induced inflammation in normal cervical epithelial cells causes malignant transformation through activation of HMGB1/Receptor for Advanced Glycation Endproducts (RAGE) axis [337]. Forkhead box protein M1 (FOXM1) is an oncogene whose knockdown enhances CD8^+^ T cell infiltration and hampers CD8^+^ T cell exhaustion as well. FOXM1 expression suppresses IL-1β/−18, potentially through NLRP3 inactivation [338]. In breast cancer, CAFs-driven NLRP3 and IL-1β drive tumor growth and metastasis by orchestrating an immune-suppressive microenvironment. This transformation involves an increased presence of myeloid cells (CD11b^+^Ly6C^high^Ly6G^−^) in the tumor, driven by the NLRP3. IL-1β enhances the presence of the granulocytic fraction (CD11b^+^Ly6G^+^Ly6C^low^) in the initial tumor as well as the monocytic fraction (CD11b^+^Ly6C^high^Ly6^G−^) in metastatic tumors [339]. NLRP3 upregulates the expression of CCL2, CCL5, CXCL5, and CXCL12, leading to increased metastasis through recruitment of MDSCs and a dynamic shift of macrophages into the anti-inflammatory phenotype [340]. Conversely, NLRP3 activation exhibits anti-tumor effects in CRC liver metastasis via promoting NK cells, which underscores the unique characteristics of malignancies [341]. Positive IL-17A protein expression, induces mitochondrial dysfunction and triggers ROS accumulation, leading to pyroptosis via the NLRP3/caspase-4/GSDMD axis [342]. IL-18 deficiency due to NLRP3 loss can impair IFN-γ production as well as STAT1 activation, thereby increasing tumor burdens in CRC [343]. It should be noted that gut microbiota-mediated activation of NLRP3/GSDMD ignites colorectal tumorigenesis and inhibition of NLRP3 might be therapeutic in this context [281].

### Specific pyroptosis components

Fukuda et al. found that AIM2 promotes tumor growth in melanoma mouse models by elevating Treg cells and reducing CD4^+^ effector T cells, with no notable changes in CD4^+^ or CD8^+^ T cells and DCs. Additionally, AIM2-deficient DCs enhance the infiltration of tumor antigen-specific CD8^+^ T cells into melanoma tumors via CXCL10. AIM2 is essential for producing IL-1β and IL-18 that encourage the accumulation of Treg cells and tumor progression in vivo [344]. Overexpression of AIM2 through STAT1/NF-κB pathway is associated with cancer progression, radioresistance, and increased PD-L1 expression, thus upregulated AIM2 predicting favorable response for ICI therapy [345]. Inhibition of AIM2 is speculated to restrain cell proliferation by blocking Wnt/β-catenin and EGFR/Ras/MEK signaling in glioma [47].

**Fig. 4:**
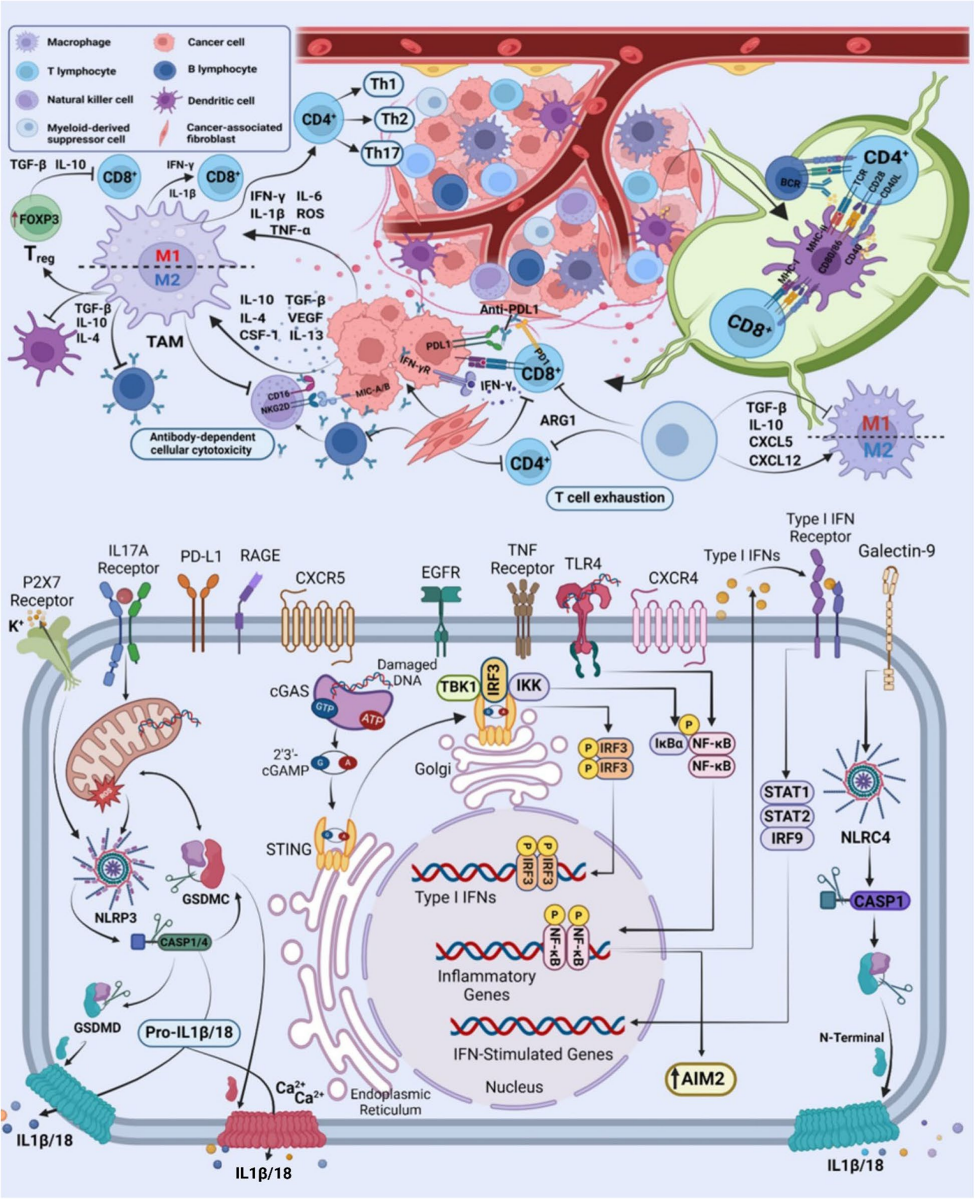
Pyroptosis-mediated remodeling of the tumor immune microenvironment. This figure shows immune interactions within the tumor microenvironment (TME) and lymph node, focusing on pyroptosis components’ dual roles in cancer immunity. In the lymph node, dendritic cells (DCs) activate B cells, T helper (CD4 +), and cytotoxic (CD8 +) T cells. M1 macrophages enhance CD4 + and CD8 + responses, while M2 macrophages, via TGF-β, IL-10, and IL-4, suppress DCs, B cells, NK cells, and activate regulatory T cells (Tregs) to further inhibit CD8 + T cells. Myeloid-derived suppressor cells (MDSCs) and cancer-associated fibroblasts (CAFs) reinforce this immunosuppression. Pyroptosis pathways affect immune activity: overexpression of gasdermin E (GSDME) triggers cGAS-STING-IFNβ signaling, promoting CD8 + proliferation, while gasdermin A (GSDMA) aids immune evasion, correlating with poor response to anti-PD-L1 therapy

Regarding the role of NLRC4, Domblides et al. showed that only the loss of NLRC4 in tumor cells, but not in stromal cells, is strongly linked to lower immune cell infiltration, particularly DCs, CD4^+^ and CD8^+^ T cells [346]. Sim et al. revealed a positive association between the the T-cell Immunoglobulin and Mucin-domain containing-3 (TIM-3)/Galectin-9 (Gal-9) axis and NLRC4, which increases with WHO glioma grade. Notably, TIM-3/Gal-9 upregulates the expression of NLRC4 and caspase-1 but does not trigger IL-1β secretion in glioma [347]. Of note, Overexpression of GSDMA plays a key role in immune evasion in glioma and is positively correlated with poor anti-PD-L1 therapy outcomes and overall survival. Knockdown of GSDMA can enhance the infiltration of CD8^+^ T and Treg cells, which could be considered in immunotherapy [348]. In the context of NSCLC, overexpression of GSDME leads to mitochondrial damage and activates the cGAS-STING-IFNβ signaling pathway, which promotes CD8^+^ T cell proliferation and results in favorable anti-cancer effects [349] (Table 2). It has been shown that IFN-γ and TNF-α can stimulate GSDMB expression. this suggests a potential synergy between cytokine production and cytotoxic mechanisms, working together to induce GSDMB-mediated pyroptosis [137] (Fig. 4).

### Key determinant of immune fate

While Table 2 summarizes the pro- and anti-tumor roles of major pyroptosis components across cancers, a mechanistic understanding of why the same pathway can lead to divergent immune outcomes remains limited. This sub-section aims to highlight key determinants, such as cancer type, ncRNA regulation, tumor stage, genetic background, microbiome state, and the immune contexture of the TME that may govern whether pyroptosis promotes antitumor immunity or immune suppression.

Tumor microenvironments differ across malignancies, which explains why the same pyroptotic components may exhibit pro- or anti-tumor effects (Table 2). As mentioned earlier, in hepatocellular carcinoma, early- versus late-stage tumors show divergent caspase-8 responses, reflecting TME plasticity [82]. Genetic and epigenetic factors, such as the methylation status of GSDME, influence pyroptosis induction and can convert immunosuppressive “cold” tumors into “hot” tumors responsive to immunotherapy, serving as potential biomarkers of therapy sensitivity [249]. Notably, gut microbiota further modulates pyroptotic outcomes. It has been shown in colorectal cancer that NLRP3 activation can enhance antitumor immunity by promoting NK cell activity and IL-18/IFN-γ signaling, whereas microbiota-driven dysregulation of NLRP3/GSDMD may instead support tumorigenesis through disrupted signaling [281]. Collectively, these observations indicate that the net effect of pyroptosis is dictated by a complex interplay of intrinsic tumor characteristics, immune contexture, and extrinsic microbial cues.

It should be noted that GSDME usually promotes antitumor responses, yet in glioma, sublytic activation can yield paradoxical effects. CAR T cell-induced pyroptosis further illustrates this complexity: granzyme B activates caspase-3 and GSDME in tumor cells, which triggers caspase-1-dependent GSDMD in macrophages, causing cytokine release syndrome. Here, pyroptotic severity depends more on CAR T cytotoxicity than baseline CD8 + T cells [135].

Of note, the regulation of pyroptosis by ncRNAs represents a critical switch that can polarize the TME toward either immune activation or suppression (Table 3). For instance, upregulation of oncogenic lncRNAs like XIST or SNHG7 to suppress NLRP3 may not just promote cell survival but also actively foster an immunosuppressive TME by preventing the release of immunostimulatory cytokines. In terms of miRNA, miR-214 has been shown to enhance pyroptosis by activating caspase-1 and elevating IL-1β/−18 secretion. Once released into the TME, these cytokines promote DC maturation, recruit antigen-specific CD8^+^ and Th1 CD4^+^ T cells, and suppress Treg differentiation, collectively fostering a robust antitumor immune response [350, 351]. Another example is lncRNA NEAT1 that its downregulation can impair T cell responses and promote apoptosis via the miR-125/mast cell expressed membrane protein 1 (MCEMP1) axis, while its upregulation enhances pro-inflammatory signaling by increasing CXCL8 and TNF-α expression and activating the MAPK pathway [352]. These illustrate how ncRNA-driven modulation of pyroptosis transcends the tumor cell itself and acts as a lever to reprogram the immune ecosystem.

As a novel and testable hypothesis, we propose defining a “functional cut-off” for pyroptosis components based on gasdermin expression (GSDME/GSDMD), cytokine profiles, and the magnitude of cell death (sublytic vs. lytic), along with cancer stage and cellular type to predict whether pyroptosis elicits anti-tumor or immunosuppressive effects in a given tumor. These thresholds can be determined through integrated molecular measurements (RNA/protein), real-time assessment of pyroptotic intensity (live-cell imaging/LDH release), and TME immune profiling (CD8⁺ T cells, Tregs, NK cells, macrophages), combined with bioinformatic modeling, providing a mechanistically guided framework for optimizing pyroptosis-based combination therapies.

## Therapeutic potential of pyroptosis

### Nanomedicine-enabling pyroptosis

A substantial body of research now supports the idea that the dysregulation of pyroptosis can impede pathogen clearance, compromise the effectiveness of adaptive immune defenses, and influence all phases of carcinogenesis. The current focus on leveraging nanomedicine to stimulate pyroptosis for more effective tumor treatment has gained increasing attention. Besides that, the dual nature of pyroptosis, serving either pro-cancer or pro- “host”, underscores the need for thorough research to maximize its potential for advancements in diverse cancer therapies [353].

Magnetic fluid hyperthermia (MFH) is an innovative technique that combines magnetic nanoparticles with an alternating magnetic field (AMF), resulting in localized hyperthermia within tumors, all the while overcoming the limitations tied to penetration depth. An in vitro experiment was conducted to show that cell death caused by MFH does not rely on the caspase-3 mediated apoptosis, but rather closely dependent on the Cathepsin-B and caspase-1 mediated pyroptosis. Magnetic Fe_3_O_4_ and temozolomide were encapsulated within a well-designed framework known as temperature-sensitive liposomes (Fe/TMZ-TSL), allowing for precise heating of glioblastoma cells to around 42 °C, while the normal tissues were preserved from any damage in the presence of AMF. The elevation of temperature led to the transient opening of blood–brain barrier (BBB), enabling the controlled release of drugs and an increased in the production of free radicals. Following exposure to Fe/TMZ-TSL + AMF treatment, showed a substantial escalation in cell mortality in both U87 and U251 cell lines (48% and 56%, respectively). Moreover, the proteins associated with pyroptosis (NLRP3, caspase-1, and GSDMD) displayed significant upregulation, while the apoptotic protein (caspase-3) underwent a contrasting downregulation [354]. Fe₃O₄ magnetic nanoparticles demonstrated excellent biocompatibility, with cytotoxicity graded between 0–1 in fibroblast assays, no hemolytic or genotoxic effects, and an acute LD₅₀ of 8.39 g/kg in vivo. Although these findings confirm a favorable safety margin, Fe₃O₄ nanoplatforms are still at the preclinical stage. However, the clinical approval of SPION-based agents such as Ferumoxytol for MRI highlights their scalable synthesis, predictable pharmacokinetics, and strong translational promise for future pyroptosis-driven cancer therapies [355–357].

Zhou et al. in their study, developed FeSO_4_ nanoparticles that rapidly ionized into Fe^2+^ under slightly acidic conditions. These ions catalyzed the conversion of H_2_O_2_ into the highly toxic •OH species, thereby inducing iron-induced oxidative stress. This process led to the oxidation and oligomerization of translocase of outer mitochondrial membrane 20 (Tom20). Meanwhile, Bax, a pro-apoptotic protein, translocated into the mitochondria to facilitate the release of cytochrome c. As a result, this process triggered the activation of both caspase-3 and caspase-9, finally causing pyroptotic cell death via the cleavage of GSDME [358]. Also, Ploetz et al. synthetized a novel liposome-encapsulated metal organic frameworks (Lip-MOFs) system, which included a core made of 1,2-dileoyl-snglycero-3-phosphocholine loaded with Fe^3+^ ions and trimesic acid-based MOFs. Cancer cells efficiently endocytosed the Lip-MOFs and liberated Fe^3+^ ions in the presence of acidic conditions. Subsequently, the secretion of Fe^3+^ triggered the activation of caspases, which in turn led to the cleavage of GSDMD and lastly occurrence of pyroptosis [359].

It has been well-established that exposing cells to a hyperosmotic environment, where the osmolarity of extracellular sodium ions is low, triggers cellular swelling, disruption of the membrane cytoskeleton, and may lead to cell death [360]. Inspired by this, Li et al. constructed glutathione (GSH)-responsive virus-inspired hollow mesoporous tetrasulfide-organosilica-modified NaCl nanocrystals, denoted as NaCl@ssss-VHMS. This novel approach allowed for the gradual in situ degradation of these nanocrystals into Na^+^ and Cl^−^ ions and also resulted in a substantial increase in intracellular osmolality, independent of cell transport proteins. The mechanism of action involved the disruption of endo/lysosomes, which then triggered the liberation of cathepsin B, causing the activation of pyroptosis via NLRP3/caspase-1 signaling pathway. In addition, the combined effects of ROS and osmotic pressure notably decreased the levels of tumor growth [361]. On scalability, the core silica/tetrasulfide chemistry is adaptable to large-scale sol–gel synthesis, but achieving consistent pore architecture, NaCl loading, degradation kinetics, and batch reproducibility presents a manufacturing challenge. To our knowledge, currently, this platform is in the preclinical phase, with no reports of clinical translation. Regarding biosafety, NaCl@VHMS systems show inherently low chemical toxicity since they are composed of physiological ions (Na⁺ and Cl⁻). Any observed cytotoxicity at high doses is mainly related to osmotic stress rather than intrinsic material toxicity [361].

Intriguingly, a huge concentration has been focused on the selective delivery of specific nanoparticles toward endosomes and lysosomes, leading to the induction of diverse forms of PCD, such as ferroptosis, necroptosis, autophagy, and pyroptosis [362]. Endosomes typically have PH levels between 5.5 to 6.8, while lysosomes maintain an optimal PH of around 5.0 [363]. Recently, scientists have engineered a set of acid-responsive nanophotosensitizers (ANPS) with mPEG-bP(R1-Rr2) as the fundamental building block. These ANPSs show a notable shift in PH from 5.3 to 6.9, effectively covering the complete spectrum associated with the maturation of endosomes. The advanced ultra-PH-sensitive nanomaterials were able to start an extensive GSDME-derived pyroptotic pathway, by activating phospholipase C (PLC). In contrast, the pyroptotic process was notably hindered in late lysosomes (PH: 5.5–4.5) and endosomes (PH: 6.1–5.5), due to the lack of PLC and the deactivation of cathepsin B during the late stages of endosomes and lysosomes [364]. At present, this nanoparticle platform remains in the preclinical stage, and while it demonstrates promising efficacy, its large-scale manufacturing and clinical translation may face challenges related to reproducible synthesis, stability, and efficient delivery to target tissues.

Many therapeutic drugs trigger caspase-3-derived apoptosis, effectively eliminating cancer cells and laying the pivotal groundwork for pyroptosis as well, a process regulated by GSDME. However, a major challenge arises because most cancer cells do not express the necessary GSDME protein for caspase-3-driven pyroptosis, as the DFNA5 gene is often hypermethylated [365]. As discussed in "The role of pyroptosis in cancers" section, the DFNA5 gene encoding GSDME is frequently hypermethylated and silenced in multiple cancers, limiting the ability of caspase-3 to execute pyroptosis. Here, the use of decitabine provides a direct solution by reversing DFNA5 hypermethylation and restoring GSDME expression, thereby re-sensitizing tumors to pyroptosis induction. To overcome this challenge, a liposome-based nanocarrier was synthetized, in which DNA methyltransferase decitabine, a cytosine analogue known for its ability to reactivate silenced genes, along with the chemotherapy drug cisplatin (Lipo-DDP), were encapsulated. After three hours following intravenous injection, Lipo-DDP rapidly aggregated in the tumor tissue, and fluorescence signals persisted at the tumor site for an impressive 36 h post-injection. Moreover, the collaborative treatment with Lipo-DDP and decitabine dramatically enhanced the maturation of dendritic cells (DCs) and activated CTLs in the tumor microenvironment. These findings underscore the consistent delivery of essential therapeutic doses to induce pyroptosis as well as potential efficiency of this combined treatment in triggering the immunological responses in a living organism [366]. Liposomal nanocarriers exhibit excellent biocompatibility and scalability, as several formulations such as pegylated liposomal doxorubicin (Doxil®) and liposomal cisplatin have advanced to or gained FDA approval. These clinically validated systems demonstrate reduced systemic toxicity and predictable pharmacokinetics [367, 368]. Although the co-encapsulated Lipo-DDP formulation remains at the preclinical stage, its design builds on well-established liposomal platforms, underscoring strong translational potential for safe and controllable pyroptosis-based cancer therapies. Their success in targeted delivery in neurological models highlights the potential of liposomal platforms for precise drug delivery in cancer [369, 370].

### Chemotherapy-induced pyroptosis agents

Chemotherapy, known as a major part of cancer treatment protocols, is of paramount importance treatment modality across various cancer types and stages [371]. Up to now, several chemotherapeutic drugs and agents have been found that are able to induce tumor cell pyroptosis, including 5-FU, cisplatin, paclitaxel, lobaplatin, etc. [372]. Wang et al. reported that treatment of MKN-45 and SGC-7901 cell lines with 5-FU can change the route of programed cell death from caspase 3-dependent apoptosis to pyroptosis [262]. Moreover, lobaplatin as a third-generation of platinum-based anti-neoplastic agent, induces GSDME-dependent pyroptosis in nasopharyngeal carcinoma (NPC) through caspase-3 activation and modulation of cellular Inhibitor of Apoptosis Protein 1/2 (cIAP1/2) proteasomal degradation [373]. The underlying caspase-3/GSDME pathway is also evident in lobaplatin-treated CC cells [374]. Other than this, in CRC cells, lobaplatin induces pyroptosis by recruiting of caspase-3/9 through ROS/JNK/BAX-mitochondrial apoptotic signaling pathway. Interestingly, it has been shown that knockout of GSDME could reduce inflammation induced by lobaplatin in CRC [375]. In A549 lung cancer cells, Cheng Zhang et al. demonstrated that between paclitaxel and cisplatin, cisplatin has more potential to induce caspase-3/GSDME-dependent pyroptosis. It suggests that cisplatin may be a preferred option rather than paclitaxel [376]. It has been determined that four antibiotics, actinomycin-D, epirubicin, daunorubicin, and doxorubicin, induce the upregulation of nPD-L1, GSDMC, and caspase-8, resulting in pyroptosis in cancer cells [55].

In addition to the aforementioned drugs, various compounds and drugs derived from natural resources can also stimulate pyroptotic cell death. Alpinumisoflavone (AIF), an isoflavone derived from *Derris Eriocarpa*, stimulates GSDME in HCC by inducing NLRP3 and recruiting caspase-3. AIF suppresses the migration, invasion, and proliferation of Huh7 and SMMC 7721 cells. ATG5 knockdown restrains autophagy and increases the level of AIF-induced pyroptosis [377]. Berberine, an isoquinoline alkaloid elevates caspase-1 expression in a dose-dependent manner, effectively inducing pyroptosis to inhibit the viability, migration, and invasion ability of HepG2 cells [378]. Additionally, metformin, a biguanide derivative, induces GSDMD-mediated pyroptosis by targeting miR-497/proline-, glutamic acid-, and leucine-rich protein 1 (PELP1) axis. This process can be abrogated by overexpression of PELP1 (Table 3) [182]. Euxanthone, a xanthone derivative and found in *Polygala caudata*. suppresses cell proliferation and migration of HCC in a caspase-2-dependent manner [379]. In NSCLC, Li et al. revealed that piperlongumine (PL) analogue L50377 restrains cell growth, promotes pyroptosis (probably via NF-κB suppression) as well as apoptosis by provoking ROS generation [380]. In regard to glioma, several small molecules and drugs, such as galangin (through caspase-3/GSDME axis) [381], benzimidazole (through NF-kB/NLRP3/GSDMD axis) [382], kaempferol (through GSDME and ROS) [383], and AT7519 (through caspase-3 cleavage of GSDME) [384] have shown the capability of inducing pyroptosis.

Combination chemotherapy is a common alternative approach used in clinical treatment for those cancers that show resistance issues to single drug therapy. BIX-01294, in conjunction with cisplatin, elicits pyroptosis through an autophagy-dependent mechanism along the Bax/caspase-3/GSDME axis. This combined strategy not only induces pyroptosis but also amplifies the chemosensitivity in NPC. Also, the knocked out *ATG5* gene can block both pyroptosis and autophagy [385]. The synergistic application of low-dose cisplatin and BI2536 effectively attenuates the viability of GSDME-positive esophageal squamous cell carcinoma via inducing caspase 3 pyroptosis pathway [386].

Oxaliplatin combined with GW4064 abolishes the growth and colony formation of CRC cells. Further explorations showed that GW4064 enhances cellular sensitivity to oxaliplatin by instilling Bax/caspase 3/GSDME pyroptosis axis [387]. In melanoma cells, it has been shown that a v-Raf murine sarcoma viral oncogene homolog B1 (BRAF)- mitogen-activated protein kinase kinase (MEK) inhibitor agent can induce pyroptosis by triggering GSDME, leading to elevated T-cell infiltration and immune system responses. Noteworthy, in immunodeficient GSDMD-positive mice, the BRAF-MEK inhibitor is unable to induce pyroptosis and subsequent elimination processes of melanoma. This underscores that immune system reactions are required for inducing pyroptosis via the GSDME-dependent pathway [387]. The reader is referred to Table 4 for a classified list of pyroptosis-inducing compounds and further detailed information.

**Table 4:** Pyroptosis-inducing compounds grouped by mechanism

Mechanism	Compounds	Cell line/Animal model	Function	Mechanism of Action	Cancer Type	References
Caspase-3/GSDME Activators	5-Fluorouracil; Paclitaxel; Lobaplatin; Apoptin; Trimethylamine N-oxide; Dihydroartemisinin; Doxorubicin; Piperlongumine analogue L50377; Galangin; Simvastatin; Dasatinib; Osthole; Oxaliplatin (± GW4064); Miltirone; Nitidine chloride; AT7519; BI2536; Kaempferol; CDK7 inhibitors	Various gastric, ovarian, nasopharyngeal, colorectal, breast, esophageal, glioblastoma, lung, neuroblastoma models	↓Cell viability, ↓tumor growth, ↓proliferation	Caspase-3 cleavage of GSDME switches apoptosis to pyroptosis, often mediated by ROS, mitochondrial apoptosis, Bax/caspase-9 activation, or stress pathway modulation	Gastric, ovarian, nasopharyngeal, colorectal, breast, glioblastoma, esophageal, lung, neuroblastoma, melanoma	[262, 373, 375, 384, 388–400]
Caspase-1/GSDMD (Canonical Inflammasome Pathway)	Cisplatin; Secoisolariciresinol diglucoside; Cucurbitacin B; Ophiopogonin B; Nobiletin; Polyphyllin VI; Berberine; Benzimidazoles; Kaempferol (via NLRP3); Simvastatin	TNBC, colorectal, lung (NSCLC), breast, ovarian, glioblastoma, gastric, hepatocellular models	↓Proliferation, ↓migration, ↓tumor growth	NLRP3/caspase-1 activation, ROS-mediated NF-κB signaling, inflammasome assembly leading to IL-1β/IL-18 release and GSDMD cleavage	Breast, colorectal, lung, glioblastoma, gastric, hepatocellular cancers	[160, 378, 382, 401–406]
Caspase-4/−5/−11 (Non-Canonical Pathway)	Bexarotene; Citric acid	Ovarian models	↓Cell viability, ↓growth	Caspase-4 or −4/−1 activation leading to GSDME/GSDMD cleavage and TXNIP/NLRP3 axis activation	Ovarian cancer	[407, 408]
Caspase-8/GSDMC Activators	Dihydroartemisinin; Doxorubicin; Epirubicin; Daunorubicin; Actinomycin-D; TNF-α/PD-L1 axis	Breast, esophageal models	↓Cell viability, ↓proliferation	Caspase-8 cleavage of GSDMC switches apoptosis to pyroptosis; often enhanced by PD-L1/STAT3 signaling	Breast cancer, esophageal squamous cell carcinoma	[55, 129, 390, 391]
Other Gasdermin Activators	HGS-ETR1/2 antibody	HCC models	↓Tumor growth, ↓cell viability	Antibody induces GSDME-mediated pyroptosis through DR4/5 signaling, AKT inhibition, and caspase-3 activation	Hepatocellular carcinoma	[300]
Indirect/Other Mechanisms	Resveratrol; Metformin; Alpinumisoflavone; Euxanthone; BXCL701 (DPP inhibitor)	Glioblastoma, hepatocellular, pancreatic ductal adenocarcinoma models	↓Cell viability, ↓tumor growth, ↑immune infiltration	Resveratrol: JAK2/STAT3/NLRP3 suppression; Metformin: miR-497/PELP1 or FOXO3/NLRP3 axis activation; Alpinumisoflavone: NLRP3 + caspase-3/GSDME dual activation; Euxanthone: caspase-2-dependent pyroptosis; BXCL701: DPP8/9 inhibition → NLRP1/CARD8 inflammasomes	Glioblastoma, HCC, esophageal, pancreatic cancers	(182, 409–411)

### Radiation therapy

Radiotherapy, a common approach in cancer treatment, is used by roughly 50% of all patients [412, 413]. The major challenges encountered with radiotherapy include tumor resistance to irradiation and, conversely, the hyperactivation of signaling pathways that induce inflammatory components, leading to extensive injury to normal cells [414]. This can further alter the metastasis, progression, migration, and invasion traits of malignancies. To address these challenges, rational regulation of pyroptosis signaling pathways through combination therapy approaches offers promising avenues. Thus, the impact of pyroptosis on radiotherapy can vary, either enhancing or reducing its effectiveness, depending on which signaling pathways are activated.

Cao et al. demonstrated a dose- and time-dependent cleavage of GSDME following irradiation, with pyroptosis induced by various radiation modalities. The combination of chemotherapy (such as cisplatin, decitabine, or etoposide) and irradiation markedly promoted pyroptosis via the caspase-3/caspase-9/GSDME pathway. Notably, GSDME-overexpressing tumors in BALB/c mice exhibited enhanced tumor suppression, characterized by elevated CTLs and cytokine release. These findings underscore the immunogenic potential of radiation-induced pyroptosis, offering valuable insights to maximize the therapeutic benefit of tumor radiation therapy [415]. Highlighting the role of lncRNAs, the upregulated lncRNA nuclear paraspeckle assembly transcript 1 (NEAT1) modulates radiation-induced pyroptosis by negatively regulating miR-448, but not through the activation of GSDME in CRC [165]. As summarized in Table 3, ncRNAs such as NEAT1 and miR-1290 act as key regulators of pyroptotic pathways and therapeutic response. Their dysregulation contributes to radioresistance, making them promising therapeutic targets for radiosensitization strategies.

In regard to radioresistant tumors, upregulation of miR-1290 in TNBC demonstrated resistance features by inhibiting NLRP3-mediated pyroptosis [176]. Zhou et al. found that upregulation of APE1 is responsible for radioresistance in lung adenocarcinoma. Mechanistically, APE1 suppresses pyroptosis by directly interacting with AIM2 and DDX41, leading to the inactivation of STING pathway. Therefore, APE1 inhibitors should be taken into account for enhancing tumor radiosensitivity [416].

Liu et al. observed an elevation in pyroptosis and higher activation of caspase-1 when BMDMs were exposed to radiation doses of 10 and 20 Gy. Interestingly, these effects were significantly reduced when *Nlrp3* was knocked out. In vivo, 9.5 Gy of radiation led to caspase-1 activation and mortality, but these outcomes were notably attenuated in *Nlrp3* knockout mice, suggesting that NLRP3 plays a crucial role in mediating radiation-induced pyroptosis. Therefore, targeting NLRP3 and pyroptosis emerges as a promising strategy to effectively alleviate the adverse effects of radiation therapy [417].

An unsolicited side effect of thoracic radiotherapy, known as radiation-induced lung injury (RILI), includes radiation pneumonitis and radiation fibrosis [418]. Gao et al. uncovered the promising protective role of andrographolide against RILI. In C57BL/6 mice subjected to 18 Gy thoracic irradiation, andrographolide injections notably reduced early-phase lung damage, cytokine release, inflammation, and late-phase fibrosis. Additionally, in-vitro, andrographolide suppressed the AIM2 inflammasome in radiation exposed macrophages, suggesting that inhibition of AIM2 and pyroptosis pathway may mitigate RILI [419]. Moreover, it has been shown that irradiation upregulates P65 subunit of NF-κB, which is responsible for activating both forms of PCD, including pyroptosis and ferroptosis. However, pretreatment with pyrrolidinedithiocarbamate ammonium (PDTC) is an option that significantly reduces inflammatory factors and improves tissue damage [420]. According to a recent study, upregulation of miR-223-3p halted the NLRP3/caspase-1/GSDMD pyroptosis pathway, resulting in decreased infiltration of inflammatory monocytes and reduced levels of proinflammatory factors. In vivo results also demonstrated that miR-223-3p supplementation could effectively alleviate acute RILI following irradiation (readers are referred to Table 3 for a list of ncRNAs and their pathways) [421].

Another complication associated with radiation therapy is radiation-induced enteropathy (RIE) in the treatment of abdominal malignancies, which can manifest as diarrhea, vomiting, nausea, and stomach cramps [422]. Wu et al. revealed the amelioration effects of micheliolide on RIE through the inhibition of the NLRP3/caspase-1/GSDMD signaling pathway, resulting in the downregulation of IL-1β/−18, IFN-γ, TNF- α, and TGF-β1 [423]. In addition, p-Coumaric acid inhibits AIM2, NLRP3, and caspase-1, thereby reducing oxidative stress, increasing villus height, and ultimately alleviating RIE [424]. Taken together, leveraging pyroptosis to overcome tumor radioresistance or mitigate tissue injury in the context of radiation therapy is a compelling area of research that deserves further attention.

### Immunotherapy

#### CAR-T/-NK cell therapy

In recent years, an innovative therapeutic approach called chimeric antigen receptor (CAR) cell therapy has attracted huge attentions due to its promising results in several pre-clinical experiments and clinical trials [425]. CARs are artificial receptors engineered to redirect T or NK cells, independently of the major histocompatibility complex (MHC) receptors, for the recognition and elimination of cells expressing specific antigens. Despite all advancements, CAR-T cell therapy faces significant challenges that still must be more refined. For instance, severe CAR-T cell-related toxicities, limited efficiency against solid tumors, antigen escape, resistance in B cell malignancies, immunosuppressive environment, tumor infiltration and poor trafficking are some major concerns in its application that need to be improved in the near future [426]. In this part, we aim to review CAR-T/NK cells capable of inducing pyroptosis. Although the occurrence of pyroptosis may increase immune responses, in some situations, it restrains the therapeutic effects of CAR cell therapy.

Cytokine release syndrome (CRS) is a systemic inflammatory response by the immune system against CAR-T cells, attenuating the efficacy of treatment. Liu et al. showed that pyroptosis stimulates CRS reaction during CAR-T cell therapy. They found that activated caspase-3 leads to the release of granzyme B, which then forms membrane pores via GSDME to induce pyroptosis. Following that, the active form of caspase-1 cleaves GSDMD in macrophages, inducing the secretion of cytokines such as IL-1 and IL-6, and stimulating CRS. Noteworthy, the key factor for CAR-T cells to induce pyroptosis in target cells is the amount of perforin/granzyme B used, not the presence of CD8 + T cells [135]. This phenomenon directly leverages the granzyme-mediated pathway described in "Molecular mechanisms of pyroptosis" section, where granzyme B released by CAR-T cells cleaves GSDME, triggering pyroptosis.

To overcome concerns related to CRS, Liu et al. discovered that CAR-T therapy triggers IL-1β production and change macrophage phenotype via upregulation of PD-L1 and indoleamine 2,3-dioxygenase (IDO), thereby activating the AIM2 inflammasome and the α1-AR-mediated adrenergic pathway. It suggests that CAR-T cell therapy combined with suppression of AIM2 and α1-AR may induce antitumor effects and alleviate the toxic side effects of CAR-T cells, such as CRS [427].

Lu et al. introduced an innovative chimeric costimulatory converting receptor (CCCR) in CAR-NK cells, which was capable of extensively inducing pyroptosis in lung cancer H1299 cells. This engineered receptor comprised the intracellular domain of 41BB, the intracellular and transmembrane proteins of NKG2D, and the extracellular part of PD-1. The NK-tailored CCCR could convert the negative PD1 signal into a stimulatory signal, thereby reversing immunosuppressive properties associated with PD1 [428].

**Fig. 5:**
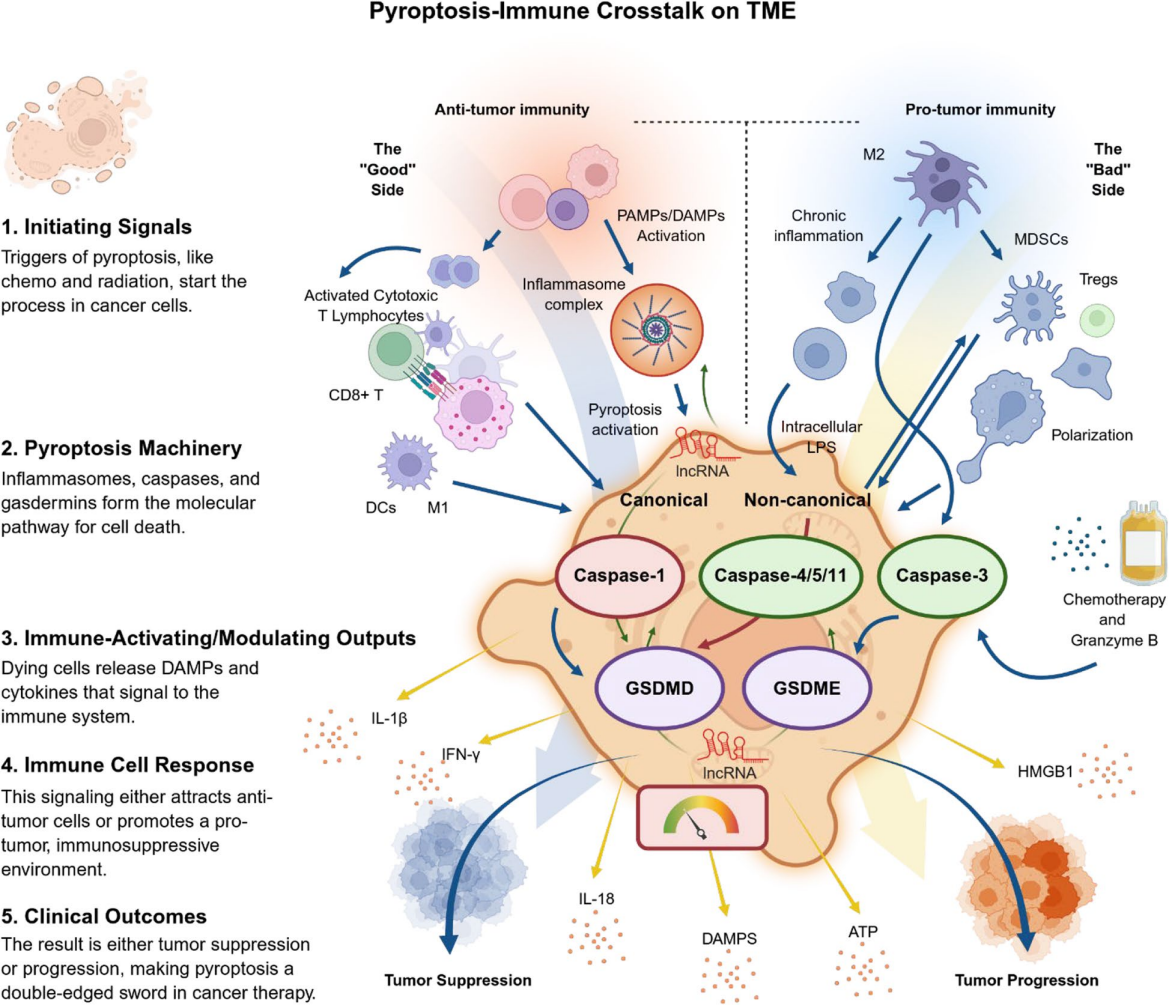
Dynamic immune crosstalk shaped by tumor cell pyroptosis. Schematic illustration of how canonical (caspase-1/GSDMD) and non-canonical (caspase-4/5/11–GSDMD, caspase-3–GSDME) pyroptosis pathways orchestrate bidirectional tumor–immune interactions. Depending on the magnitude of gasdermin activation and the regulatory influence of ncRNAs, pyroptosis can either trigger the release of proinflammatory DAMPs and promote anti-tumor immunity through NK and cytotoxic T cells, or induce chronic inflammation and recruitment of immunosuppressive cells such as M2 macrophages and MDSCs, thereby fostering tumor progression

Recent mechanistic studies clarify how pyroptotic signaling regulates immune infiltration. In CAR-T therapy, granzyme B cleaves GSDME in tumor cells, which triggers macrophage activation of caspase-1/GSDMD and leads to IL-1β/IL-6 release [10, 135] (Fig. 5). These cytokines increase vascular permeability and attract innate immune cells, but can overshoot and cause CRS when pyroptosis is unrestrained. Checkpoint blockade experiments (e.g., anti-PD-1, PD-L1) show that tumors with higher basal gasdermin expression (GSDMB, GSDME) respond better because the pyroptosis induced releases chemokines (CXCL9/10) that recruit dendritic cells and CD8⁺ T cells, amplifying the antitumor immune loop [429, 430]. Conversely, suppressors like CDC20 or methylation-silenced GSDME blunt that recruitment [431]. Hence, the magnitude of pyroptosis (sublytic vs. lytic), identity of the gasdermin, and regulatory suppression (epigenetic or ubiquitin-mediated) work together to set a threshold below which infiltration and immunity are enhanced, above which inflammation becomes suppressive or toxic. A recent study showed a novel link between GSDMB and immunotherapy responsiveness in cancer. In Her2-positive gastric cancer, Lin et al. demonstrated that a bispecific antibody targeting PD-1 and Her2 (IBI315) not only enhanced antitumor immunity but also induced GSDMB-mediated pyroptosis in tumor cells. This process promoted T-cell activation and infiltration through a positive feedback loop involving IFN-γ-driven upregulation of GSDMB and tumor lysis [432, 433]. These findings reveal that GSDMB-driven pyroptosis may act as a key effector for combination immunotherapy in GSDMB-expressing cancers.

#### Immune checkpoint inhibitors

Immune checkpoint inhibitors (ICIs), as vigorous weapons against cancer cells, have revolutionized clinical immunotherapy approaches by targeting specific receptors on immune cells such as PD-1, cytotoxic T-lymphocyte–associated protein 4 (CTLA-4), TIM3, and lymphocyte activation gene 3 (LAG-3) [434, 435]. These receptors are crucial for transducing co-stimulatory and co-inhibitory signals to affect T lymphocytes. T cells employ specific receptor-ligands to identify and tolerate normal cells, while, cancer cells adeptly exploit the immune checkpoints to evade the immune system, masquerading as normal, healthy cells [436]. Several studies have explored the combined impact of checkpoint inhibitors and pyroptosis in malignancies, which we provide them here.

Zhiwei Zhou et al. showed a synergistic tumor-suppressive role of pyroptosis in combination with PD-1 blockade in B16 melanoma and CT26 colorectal carcinoma cells. GSDMB expression, induced by tumor-infiltrating CD8^+^ T lymphocytes and immune cell-released cytokines like TNF-α and IFN-γ, establishes a positive feedback loop. Despite both GSDME and GSDMB being triggered by killer cells, GSDME appears to have stronger tumor-suppressive properties [137]. Additionally, cell division cycle 20 (CDC20), an E3 ligase upregulated in prostate cancer, downregulates pyroptosis via GSDME. Wu et al. revealed that CDC20 depletion enhances pyroptosis and anti-tumor immunity when combined with α-PD-1 therapy [431]. Another study revealed that cisplatin can enhance the effects of anti-PD-L1 therapy by activating GSDME to release IL-12 and IFN- γ in SCLC cells, while the knockout of GSDME yields a reverse result in terms of IL-12 releasing and sensitization to immunotherapy [437]. The majority of patients with HER2-positive gastric cancer ultimately develop resistance to standard anti-HER2 treatments. Li et al. developed the anti-PD-1/HER2 bispecific antibody IBI315, which demonstrated promising ani-tumor effects. This IgG1 antibody triggers GSDMB-mediated pyroptosis, leading to the activation of T cells and subsequent secretion of IFN-γ, thus creating a positive feedback loop [432]. In head and neck squamous cell carcinoma (HNSCC), Wang et al. reported that CTLA-4 blockade has the potential to stimulate tumor cell pyroptosis through STAT1/IRF1 axis, generating large amounts of chemokines and inflammatory substances such as TNF-α and IFN-γ [438].

Although, Immune checkpoint blockade therapy appears to induce a long-lasting immune response compared to chemo or targeted therapy, its low response rate, ranging from 10 to 30% in various types of cancers, has prompted extensive research to resolve these issues [439]. To optimize the efficacy of anti-PD-1 immunotherapy, Fitzgerald et al. hypothesized that combining anti-PD-1 treatment with inhibition of the serin peptidase family, dipeptidyl peptidases4 (DPP4) and DPP8/9, could effectively reinvigorate the efficacy of PD-1 blockade. They evaluated the effects of the DPP inhibitor small molecule BXCL701 (i.e., Val-broPro, Talabostat, PT100) combined with anti-PD-1 in a pancreatic ductal adenocarcinoma murine model. Upon BXCL701 treatment of mT3-2D tumors, chemoattractants CXCL9/10 as well as CXCR3^+^ cells (including CD4^+^ T, CD8^+^ T, and NK cells) were trafficked into the tumors. They also corroborated that BXCL701 + anti-PD-1 combination therapy induces a memory response against rechallenge, and tumor regression is mediated by the presence of both CD8^+^ T and NK cells; depleting these cells negates the effects of therapy [411]. It is well-established that DPP8/9 inhibitors play a role in triggering NLRP1 and CARD8 inflammasomes, leading to the activation of caspase-1 and cleavage of GSDMD [440].

#### Cancer vaccines

Cancer vaccines, as proficient tutors of immune system, can be used to educate immune cells and stimulate the production of various immune components against cancer antigens [441]. Following the administration of tumor vaccines, antigen-presenting cells (APCs) such as dendritic cells (DCs), B cells, and macrophages capture and process the antigens. These antigens are then presented on MHC class I or MHC class II molecules. Concurrently, activated T cells undergo proliferation and differentiate into effector T cells and memory T cells. The effector T cells subsequently migrate to the TME, where they target and eliminate tumor cells, triggering PCD [442]. Tumor vaccines are commonly categorized into several platforms, including cell-based, nucleic acid-based, peptide-based, and virus-based vaccines [443]. Up to now, tumor vaccines have shown favorable responses in combination with other types of cancer treatment approaches. However, tumor resistance issues have made major concerns in application of vaccines. Immunosuppressive cells (such as Tregs, MDSCs, CAFs, and M2 macrophages) and respective cytokines (such as IL-6, IL-10, VEGF, TGFβ) can avoid the activation of DC-mediated T cells and effector T cell in TME [442, 444]. Here, we provide a brief review of pyroptosis-related vaccines, highlighting their role in enhancing immune responses.

Arakelian et al. explored a novel approach to DNA vaccination by incorporating an active caspase-1 variant into the DNA expressing the antigen. In vitro, this vaccine induced IL-β release and GSDMD dependent cell death. In mice, antigen-specific DNA, combined with the active caspase-1, not only expedited CD8^+^ T cell responses but also increased survival in the face of melanoma challenges. Therefore, caspase-1, as an adjuvant immunotherapy can improve DNA vaccination through employing pyroptosis pathway [445].

He et al. manipulated tumor cells genetically to express a controlled increase in the N-terminal domain of GSDMD, inducing pyroptosis under a strict surveillance. Pyroptotic tumor cells released DAMPs and inflammatory cytokines to enhance the maturation as well as migration of BMDCs. They reported that overexpression of N-GSDMD significantly triggered both the local and systemic responses of antitumor immunity and remodeled TME, resulting in the tumor growth suppression in mice. This personalized vaccine strategy opens new fronts for immunotherapy via genetically inducing pyroptosis pathway [446].

Corripio-Miyar et al. investigated a novel vaccine delivery system using protein-coated microcrystals modified with calcium phosphate (CaP-PCMCs). The study revealed that CaP-PCMCs, which co-immobilized vaccine antigens within amino acid crystals, increased antigen-specific IgG responses in mice and induced rapid pyroptosis in immune cells from sheep, cattle, and humans. This pyroptotic response was dependent on direct cell-CaP-PCMCs contact, and neither microcrystals nor soluble calcium without CaP induced pyroptosis. These findings highlight CaP-PCMCs as a promising candidate for enhancing adaptive immunity in subunit vaccines [447].

According to a recent study by Du et al., a smart metallic Fe/Mn nanovaccine loaded with sorafenib was shown to induce immunogenic factors and activate cGAS-STING immunostimulatory pathway. Mechanistically, high levels of glutathione and low PH cause the detachment of Fe^3+^ from the construct, resulting in the robust induction of pyroptosis in synergy with sorafenib. Following this, the released immunogenic factors promote DCs and expose dsDNA, which initiates cGAS-STING pathway in synergy with Mn^2+^. Altogether, these processes induce type I IFN and reduce the immunosuppressive characteristics of liver cancer [448].

Oncolytic virotherapy is a compelling platform in cancer treatment that mostly relies on engineering viruses to invade tumor cells. A live attenuated version of the ZIKV vaccine has been shown to enter GBM tumor cells via α_v_β_5_ and/or Ax1 receptors, which in turn induce apoptosis (caspase-3) and pyroptosis (GSDMD/IL-1β). Importantly, the vaccine demonstrated the effective potential to destroy human GBM cells without causing damage to fully matured neurons or primary endothelial cells [449].

### Therapeutic strategy landscape

As mentioned earlier, various strategies, including nanoparticles, chemotherapy, immunotherapy, and ncRNA modulation, have been explored to induce pyroptosis in cancer. Each approach presents unique advantages and limitations, as well as context-dependent challenges. In this sub-section, we aim to critically assess these features, providing a structured perspective for understanding their potential applications and guiding future translational research.

Among current cancer treatment modalities, immunotherapy stands out for its ability to reprogram the immune system and induce durable antitumor responses, particularly in immunoinflammatory tumors. In contrast, its efficacy in immunosuppressive or immune-excluded tumors remains limited, with overall response rates of only 10—30%, and risks such as CRS, autoimmune complications, and, rarely, hyperprogressive disease [439, 450]. Among clinically validated strategies for addressing CRS, IL-6R blockade with tocilizumab and IL-1R inhibition with anakinra have demonstrated robust mitigation of neurotoxicity and CRS in patients, marking a clear translational success [451]. Prophylactic blockade of T cell-derived cytokines (TNF-α, GM-CSF) has shown promising results in early clinical studies, while engineering lower-affinity or conditionally activated CD3 binders remains mostly at the preclinical stage but offers a rational approach to reduce cytokine surges without impairing cytotoxicity [451, 452]. Moreover, repurposed kinase inhibitors such as janus kinase (JAK), Bruton’s tyrosine kinase (BTK), and mammalian target of rapamycin (mTOR) inhibitors have demonstrated CRS mitigation in both preclinical and limited clinical settings, highlighting their potential to fine-tune systemic inflammation [453–455].

Of note, T cell exhaustion is a critical barrier to durable antitumor immunity in immunosuppressive TME. Exhausted T cells upregulate inhibitory checkpoints such as PD-1, LAG-3, and TIM-3, limiting their cytotoxicity and proliferative capacity [456–458]. A promising avenue to overcome this involves personalized cancer vaccines designed through reverse vaccinology and immunoinformatics methods, enabling rapid identification of patient-specific neoantigens based on tumor mutations revealed by next-generation sequencing (NGS) and RNA sequencing techniques [459, 460]. Although preclinical studies show encouraging immune reactivation, these strategies are still in early development and warrant further investigation to validate safety, efficacy, and clinical feasibility. Nevertheless, they offer a compelling route to restore exhausted T cell function and transform immunologically “cold” tumors into responsive targets.

Chemotherapy, while less selective, offers broad applicability and remains a backbone for many cancers, but systemic toxicity and acquired resistance are key challenges. Nanomedicine, especially nanoparticles delivering ncRNAs or targeted agents, offers a strategy to bridge these limitations: by modulating pyroptosis regulators like NLRP3 within the TME, nanoparticles can reshape immunosuppressive TME, boost CD8^+^ T cell infiltration, and restore responsiveness to immunotherapy. This combinatorial approach highlights the potential to convert immunologically “cold” tumors into “hot” ones, enhancing the therapeutic window of checkpoint inhibitors while reducing systemic toxicity.

As summarized in Table 2, GSDME activation generally confers robust antitumor effects across most cancers, with gliomas as a notable exception where outcomes depend on sublytic versus lytic pyroptosis. In GSDME-proficient tumors, pyroptosis enhances antitumor immunity and chemosensitivity, whereas GSDME-deficient tumors require alternative cytotoxic strategies. However, as shown by Liu et al., excessive GSDME activation in CAR T cell therapy can induce CRS through bystander cell inflammation. Thus, spatially targeted activation, such as via functionalized nanoparticles may harness GSDME’s therapeutic potential while minimizing CRS risk.

Overall, pyroptosis-centered therapies are converging toward a more precise and integrated paradigm. The most promising paths forward include 1) tumor-restricted GSDME activation, which serves as a key therapeutic lever capable of amplifying antitumor immunity while mitigating CRS through spatially confined control. 2) nanoparticle–ncRNA platforms, representing a second frontier that restore silenced pyroptotic genes with molecular precision and synergize effectively with chemo- or immunotherapy; and 3) AI-assisted personalized cancer vaccines, powered by NGS-based neoantigen mapping, which offer a third route to rejuvenate exhausted T cells and reprogram the tumor immune landscape.

## Clinical relevance and translational perspectives

Pyroptosis modulation holds immense promise for cancer therapy, yet its translation to the clinic remains nascent, underscoring key challenges in harnessing this immunogenic cell death pathway. To our knowledge, searching keywords such as "pyroptosis," "inflammasome," "gasdermin," and "NLRP3" in ClinicalTrials.gov (as of September 2025) yielded no direct trials explicitly targeting pyroptosis. However, our literature review could identify a few clinical trials related to pyroptosis, which we discuss below.

Zhao et al. evaluated low-dose apatinib, alone or with melittin, in a phase II trial for anaplastic thyroid carcinoma. The study revealed a high disease control rate (88.2%), but nearly a quarter of patients discontinued treatment due to toxicity, highlighting a key translational challenge. Mechanistically, synergistic antitumor effects were mediated via activation of both caspase-1–GSDMD and caspase-3–GSDME pathways, connected through a two-way positive feedback loop, which enhanced efficacy while potentially allowing dose reduction. For researchers, the study underscores the need to strategically design combinations that exploit this dual-pathway synergy, carefully titrate dosing to avoid excessive toxicity, and integrate real-time monitoring of pathway activity to guide treatment decisions [461].

Another study by Liu et al. showed that CAR T cells trigger tumor cell pyroptosis through granzyme B–mediated activation of caspase-3 and cleavage of gasdermin E (GSDME), which then activates caspase-1–dependent GSDMD in macrophages, causing cytokine release syndrome (CRS). While this pyroptosis enhances antitumor activity, it also drives systemic inflammation, making CRS a major translational challenge. Importantly, the severity of pyroptosis depends on CAR T cell cytotoxicity rather than preexisting CD8^+^ T cells, and modulating GSDME or macrophage activity can prevent CRS in preclinical models. For future studies, researchers should carefully titrate CAR T cell doses, screen tumors for GSDME expression to anticipate CRS risk, consider co-administering agents that modulate macrophage activation to limit excessive inflammation, and monitor pyroptotic and inflammatory biomarkers in real time to guide adaptive treatment [135].

A recent study demonstrated that PARP inhibitors induce pyroptosis in ovarian cancer via a TNF–caspase-8–GSDMD/E axis, generating immunogenic cell death that remodels the tumor immune microenvironment and expands T cells specific for tumor-derived neoantigens. Mechanistically, pyroptosis depends on TNF signaling and gasdermin expression, which could serve as potential criteria for patient stratification. The release of neoantigens provides an opportunity to prime highly tumor-specific immune responses, potentially exploitable in combination with immune checkpoint inhibitors [462]. In terms of defining a stratification system, choosing the right genes for a panel (e.g., CASP9, GSDME, IL1β, TIRAP), standardizing assays, and validating thresholds across diverse patient populations are all significant challenges. Combining these molecular insights with immune profiling, cytokine measurements, and tumor characteristics could enable a multi-dimensional patient assessment, but the approach is still costly and complex [463].

Taken together, the clinical translation of pyroptosis hinges on understanding its context rather than merely demonstrating its presence. As mentioned earlier, among key regulators, GSDME stands out as a decisive switch: when present, its cleavage by caspase-3 shifts apoptosis toward pyroptosis, enhancing antitumor immunity but potentially triggering cytokine-related toxicity, as seen in CAR T cell therapy. When absent, this immunogenic pathway is blocked, allowing tumors to evade immune detection. This duality highlights that the central question is not whether to induce pyroptosis, but “when” and “to what extent” it should be triggered. A rational path forward will require integrating biomarkers, including gasdermin expression, inflammasome activity, and neoantigen load into patient stratification frameworks, enabling pyroptosis to be harnessed as a precise and controllable therapeutic strategy rather than a double-edged sword (Table 5).

**Table 5:** Key Takeaways for pyroptosis in cancer: translational and clinical insights

Aspect	Key Insights & Translational Implications
Dual role	Pyroptosis inherently carries a dual potential: it can stimulate antitumor immunity but may also drive systemic inflammation, CRS, or therapy resistance if uncontrolled. Stratifying patients by gasdermin expression, inflammasome activity, and neoantigen load allows safe, targeted activation
Cancer context	Tumor effects are context-dependent: e.g., GSDMC may promote CRC/HCC, while GSDME seems to be tumor-suppressive in CRC. ncRNAs modulate pathway activity and could guide personalized interventions
Therapeutic exploitation	Combining chemotherapy, ICIs, CAR-T, or nanoparticle-based delivery can trigger pyroptosis selectively. Monitoring gasdermin status and cytokine levels enables safer, adaptive combination strategies
Translational challenges	Risks include cytokine release (CRS), off-target tissue injury, and therapy resistance. Limited in vivo biomarkers and context-specific effects complicate clinical translation
Future directions	Multi-modal approaches (nanoparticles, ncRNA modulators, STING agonists, oncolytic viruses) and AI-guided biomarker profiling can optimize patient selection and dose timing, converting pyroptosis into a precision therapy

*Abbreviations: CRC* Colorectal cancer, *HCC* Hepatocellular carcinoma, *ICIs* Immune checkpoint inhibitors, *CAR-T* Chimeric antigen receptor T cells, *CRS* Cytokine release syndrome, *ncRNA* Non-coding RNA, *STING* Stimulator of interferon genes, *AI* Artificial intelligence

## Future perspective

Despite significant progress in decoding the mechanisms of pyroptosis and its therapeutic modulation, translating these insights into clinically meaningful outcomes remains limited. The dual nature of pyroptosis and its context-dependent effects across cancer types highlight the need for a more integrative and stage-specific approach. Therefore, future directions should focus on transforming mechanistic understanding into predictive and targetable frameworks that bridge molecular insights with therapeutic application.

As summarized in Table 2, our synthesis delineates distinct pro- and anti-tumor patterns of pyroptosis across different cancers. However, this descriptive view alone cannot guide clinical translation. To harness pyroptosis therapeutically, future studies should establish a multi-dimensional classification framework that integrates (i) tumor stage (early vs. established), (ii) immune contexture (hot vs. cold; TAM/MDSC abundance, CD8^+^ infiltration), (iii) cellular lineage/phenotype, (iv) therapy-sensitivity profile (chemo-sensitive vs. resistant), and (v) quantitative molecular biomarkers (e.g., gasdermin/caspase protein levels and GSDME promoter methylation). Such stratification will clarify why, for instance, caspase-8 exhibits opposing effects in early versus advanced hepatocellular carcinoma, and will enable predictive modeling and biomarker-guided clinical trial design. Moreover, tumor-specific mutations and GSDM expression appear to influence pyroptosis efficacy. In GSDME-proficient tumors, pyroptosis often promotes cell death and can enhance chemosensitivity, whereas in tumors with mutations or silenced GSDMs, this response may be attenuated. Accordingly, assessing tumor GSDM status could help guide patient selection, and targeted approaches.

By integrating the insights from Tables 2 and 4, it becomes evident that among pyroptosis mediators, GSDME stands out as the most consistently anti-tumor component across multiple cancer types, with diverse upstream activators capable of harnessing its tumor-suppressive potential. It is noteworthy that directly inhibiting upstream regulators such as NLRP3 or caspase-8 may not always be optimal, as these nodes orchestrate multiple signaling cascades essential for immune balance and tissue homeostasis. Instead, focusing on downstream effectors where the cellular fate is ultimately determined offers a more refined therapeutic window. The extensive regulatory network of ncRNAs uncovered in this review (Table 3) positions them as a promising strategy to modulate pyroptosis precisely, enabling selective activation or silencing of gasdermins and caspases. Such precision control could be leveraged in combination strategies, for example by co-delivering selected ncRNAs with chemotherapeutic agents via targeted nanocarriers to sensitize tumors through GSDME activation. Among nanoparticles, magnetic fluid hyperthermia (MFH) and liposome platforms have advanced the furthest toward clinical translation, whereas emerging nanoparticles designed to exploit pH or osmotic perturbations in the TME represent promising strategies but remain at an early preclinical stage.

Notably, Nanoparticle-guided pyroptosis modulation offers a strategic solution to a fundamental challenge in cancer therapy: balancing potent antitumor effects with minimal collateral damage. By confining pyroptotic activation to the tumor, nanoparticles can enhance immune infiltration and cytotoxicity in immunosuppressive TMEs, while sparing normal tissues. Conversely, in hyperinflammatory contexts such as CRS, nanoparticles can be engineered to co-deliver cytokine and gasdermin inhibitors (e.g., anti-IL-6, ant-GSDME, or anti-TNF), dampening systemic inflammation without compromising efficacy. This spatially and contextually tailored approach represents a compelling next frontier toward balancing efficacy and safety in immunotherapy.

Finally, this review goes beyond cataloging pyroptosis mechanisms by integrating three complementary dimensions: ncRNA-based modulators, context-dependent pro- and anti-tumor effects, and pyroptosis-inducing compounds (Tables 2, 3, and 4). Together, these tables provide a concise yet comprehensive view, highlighting how tumor type, GSDME status, and immune context dictate therapeutic outcomes. By connecting mechanistic insights with translational relevance, this synthesis frames a roadmap for precision, biomarker-guided interventions that maximize antitumor efficacy while minimizing systemic toxicity, offering researchers a practical and cohesive guide to innovate in pyroptosis-centered oncology.

## Data Availability

No datasets were generated or analysed during the current study.

## Abbreviations:

DAMP: Damage-associated Molecular Pattern
TME: Tumor Microenvironment
PCD: Programmed Cell Death
CASPs: Caspases
GSDMs: Gasdermins
NSCLC: Non-small Cell Lung Cancer
TAMs: Tumor-associated Macrophages
CAR-T: Chimeric Antigen Receptor T-cell
ICIs: Immune Checkpoint Inhibitors
LPS: Lipopolysaccharides
PAMP: Pathogen-associated Molecular Pattern
DAMP: Danger-associated Molecular Pattern
mtDNA: Mitochondrial DNA
PRR: Pattern Recognition Receptor
NLRP3: NOD-Like Receptor family, Pyrin domain containing 3
NLRC4: NOD-Like Receptor family CARD domain containing 4
AIM2: Absent in Melanoma 2
LRR: Leucine-Rich Repeat
N-GSDMD: N-terminal fragment Gasdermin D
C-GSDMD: C-terminal fragment Gasdermin D
IL: Interleukin
NF-kB: Nuclear factor kappa B
T3SS: Type III secretion system
MAVS: Mitochondrial Antiviral Signaling Protein
TLR: Toll-like receptor
NAIP: NLR family Apoptosis Inhibitory Protein
TWIK2: Tandem of pore-domains in a Weakly Inward rectifying K^+^ channel 2
VRACs: Volume-regulated Anion Channels
HMGB1: High-mobility group box 1
RIPK1: Receptor-interacting protein kinase 1
PARPi: Poly ADP-ribose Polymerase inhibitors
ELANE: ELAstase-neutrophil expressed
SpeB: Streptococcal Pyrogenic Exotoxin B
miRNA: MicroRNAs
lncRNA: Long non-coding RNAs
TMZ: Temozolomide
GBM: Glioblastoma
GNB4: Guanine nucleotide-binding protein-4
cGAS–STING: Cyclic GMP–AMP synthase – stimulator of interferon gene
EZH2: Enhancer of Zeste Homolog 2
BHB: Beta-Hydroxybutyrate
MyD88: Myeloid differentiation primary response 88
p38-MAPK: P38 mitogen-activated protein kinase
SIRT1: Sirtuin 1
CLC3: Chloride channel protein 3
SGK1: Serum/Glucocorticoid-Regulated Kinase 1
PI3K: Phosphatidylinositol 3-kinase
PDK1: 3-Phosphoinositide-dependent protein kinase-1
MCP-1: Monocyte chemoattractant protein-1
EGFR: Epidermal Growth Factor Receptor
BAK: Bcl-2 homologous Antagonist/Killer
PD-L1: Programmed Death-Ligand 1
JAK-STAT3: Janus kinase/signal transducer and activator of transcription 3
AMIGO2: Adhesion molecule with IgG-like domain 2
LCN2: Lipocalin 2
MMP-9: Matrix Metalloproteinase-9
ANGPTL4: Angiopoietin-like 4
SOD2: Superoxide Dismutase 2
SCGB3A2: Secretoglobin family 3A member 2
SDC1: Syndecan-1
MELK: Maternal Embryonic Leucine Zipper Kinase
Twist1: Twist family BHLH transcription factor 1
Snai2: Snail family transcriptional repressor 2
HDACi: Histone Deacetylase Inhibitor
TNBC: Triple-negative Breast Cancer
Hsp90: Heat Shock protein 90
Rac-1: Ras-related C3 botulinum toxin substrate 1
Cdc42: Cell division control protein 42 homolog
CDK7: Cyclin-dependent kinase 7
HIF-1α: Hypoxia-inducible factor 1-alpha
DRP1: Dynamin-related protein 1
METTL3: Methyltransferase-like 3
IGF2BP3: Insulin-like growth factor 2 mRNA binding protein 3
CagA: Cytotoxin-associated gene A
CCL19: C-C motif chemokine ligand 19
CCR7: C-C motif chemokine receptor 7
5-FU: 5-Fluorouracil
ALKBH4: AlkB Homolog 4
H3K4me3: Trimethylation of lysine 4 on histone H3
NRF2: Nuclear Factor erythroid 2-related Factor 2
CRC: Colorectal Cancer
FOXP2: Forkhead Box P2
PCNA: Proliferating Cell Nuclear Antigen
TGFBR2: Transforming Growth Factor Beta receptor 2
ERK1/2: Extracellular signal-regulated kinase 1/2
mTORC1/2: Mechanistic target of rapamycin complex 1/2
circPDIA3: Circular RNA Protein Disulfide Isomerase A3
XBP1: X-box binding protein 1
ZDHHC3/17: Zinc Finger DHHC-Type Palmitoyltransferase 3 / 17
MACC1: Metastasis-associated in colon cancer 1
S100A8: S100 calcium binding protein A8
NFS1: Cysteine desulfurase
HDAC2: Histone deacetylase 2
H3K27ac: Histone H3 Lysine 27 acetylation
BRD4: Bromodomain-containing protein 4
Syk: Spleen tyrosine Kinase
TBK1: TANK-binding Kinase 1
IRF3: Interferon Regulatory Factor 3
HCC: Hepatocellular carcinoma
NEK7: NIMA related kinase 7
Sorcin: Soluble resistance-related calcium-binding protein
SIRT1: Sirtuin 1
SNHG7: Small nucleolar RNA host gene 7
HBX: Hepatitis B virus X protein
HMGCR: 3-Hydroxy-3-methyl-glutaryl-coenzyme A reductase
BRCC36: BRCA1/BRCA2-containing complex 3
RNF20: Ring finger protein 20
DDX3X: DEAD-box helicase 3 X-linked
E2F1: E2 promoter-binding transcription factor 1
DR4/5: Death receptor 4 / 5
CPA4: Carboxypeptidase A4
PP2A: Protein phosphatase 2
CC: Cervical Cancer
HPV: Human papillomavirus
KIF23: Kinesin family member 23
CDH3: Cadherin-3
MALAT1: Metastasis Associated Lung Adenocarcinoma Transcript 1
LRRC75A-AS1: Leucine rich repeat containing 75A antisense RNA 1
IGF2BP1: Insulin-like growth factor 2 mRNA-binding Protein 1
SYVN1: E3 ubiquitin-protein ligase synoviolin
SIX1: Sine oculis homeobox homolog 1
IFI16: Interferon gamma-inducible protein 16
TRIM21: Tripartite motif-containing protein 21
IRF6: Interferon Regulatory Factor 6
OC: Ovarian Cancer
NINJ1: Ninjurin 1
PFKFB3: 6-Phosphofructo-2-Kinase/Fructose-2,6-Biphosphatase 3
HOTTIP: HOXA transcript at the distal tip
ASK1: Apoptosis signal-regulating kinase 1
GBP5: Guanylate-binding protein 5
CENPM: Centromere Protein M
TRPM2: Transient Receptor Potential Melastatin 2
MDSCs: Myeloid-derived Suppressor Cells
CAFs: Cancer-associated Fibroblasts
ECM: Extracellular Matrix
MSCs: Mesenchymal stem cells
CTLs: Cytotoxic T lymphocytes
TCR: T cell receptor
NK Cells: Natural killer cells
Treg: Regulatory T cells
RAGE: Receptor for advanced glycation end products
FOXM1: Forkhead box protein M1
TIM-3: T-cell immunoglobulin and mucin-domain containing-3
Gal-9: Galectin-9
Tom20: Translocase of outer mitochondrial membrane 20
GSH: Glutathione
cIAP1/2: Cellular inhibitor of apoptosis protein 1/2
PELP1: Proline-, glutamic acid-, and leucine-rich protein 1
BRAF: V-raf murine sarcoma viral oncogene homolog B1
MEK: Mitogen-activated protein kinase kinase (MAP2K1/2)
RILI: Radiation-induced Lung Injury
NEAT1: Nuclear paraspeckle assembly transcript 1
APE1: Apurinic/apyrimidinic endonuclease 1
CRS: Cytokine Release Syndrome
NKG2D: Natural killer group 2 member D
CTLA-4: Cytotoxic T-lymphocyte–associated protein 4
CDC20: Cell Division Cycle 20
DPP4: Dipeptidyl Peptidases 4
APCs: Antigen-presenting Cells
